# Differences in the intrinsic chondrogenic potential of human mesenchymal stromal cells and iPSC‐derived multipotent cells

**DOI:** 10.1002/ctm2.1112

**Published:** 2022-12-19

**Authors:** Shiqi Xiang, Zixuan Lin, Meagan J. Makarcyzk, Kanyakorn Riewruja, Yiqian Zhang, Xiurui Zhang, Zhong Li, Karen L. Clark, Eileen Li, Silvia Liu, Tingjun Hao, Madalyn R. Fritch, Peter G. Alexander, Hang Lin

**Affiliations:** ^1^ Department of Orthopaedic Surgery University of Pittsburgh School of Medicine Pittsburgh Pennsylvania USA; ^2^ Department of Orthopaedics The Second Xiangya Hospital Central South University Changsha Hunan PR China; ^3^ Department of Bioengineering University of Pittsburgh Swanson School of Engineering Pittsburgh Pennsylvania USA; ^4^ Osteoarthritis and Musculoskeleton Research Unit, Faculty of Medicine Chulalongkorn University, King Chulalongkorn Memorial Hospital, Thai Red Cross Society Bangkok Thailand; ^5^ Department of Pathology University of Pittsburgh School of Medicine Pittsburgh Pennsylvania USA; ^6^ McGowan Institute for Regenerative Medicine University of Pittsburgh School of Medicine Pittsburgh Pennsylvania USA

**Keywords:** cartilage regeneration, chondrocyte hypertrophy, chondrogenesis, induced pluripotent stem cells, mesenchymal stromal cells, Smad

## Abstract

**Background:**

Human multipotent progenitor cells (hiMPCs) created from induced pluripotent stem cells (iPSCs) represent a new cell source for cartilage regeneration. In most studies, bone morphogenetic proteins (BMPs) are needed to enhance transforming growth factor‐β (TGFβ)‐induced hiMPC chondrogenesis. In contrast, TGFβ alone is sufficient to result in robust chondrogenesis of human primary mesenchymal stromal cells (hMSCs). Currently, the mechanism underlying this difference between hiMPCs and hMSCs has not been fully understood.

**Methods:**

In this study, we first tested different growth factors alone or in combination in stimulating hiMPC chondrogenesis, with a special focus on chondrocytic hypertrophy. The reparative capacity of hiMPCs‐derived cartilage was assessed in an osteochondral defect model created in rats. hMSCs isolated from bone marrow were included in all studies as the control. Lastly, a mechanistic study was conducted to understand why hiMPCs and hMSCs behave differently in responding to TGFβ.

**Results:**

Chondrogenic medium supplemented with TGFβ3 and BMP6 led to robust in vitro cartilage formation from hiMPCs with minimal hypertrophy. Cartilage tissue generated from this new method was resistant to osteogenic transition upon subcutaneous implantation and resulted in a hyaline cartilage‐like regeneration in osteochondral defects in rats. Interestingly, TGFβ3 induced phosphorylation of both Smad2/3 and Smad1/5 in hMSCs, but only activated Smad2/3 in hiMPCs. Supplementing BMP6 activated Smad1/5 and significantly enhanced TGFβ’s compacity in inducing hiMPC chondrogenesis. The chondro‐promoting function of BMP6 was abolished by the treatment of a BMP pathway inhibitor.

**Conclusions:**

This study describes a robust method to generate chondrocytes from hiMPCs with low hypertrophy for hyaline cartilage repair, as well as elucidates the difference between hMSCs and hiMPCs in response to TGFβ. Our results also indicated the importance of activating both Smad2/3 and Smad1/5 in the initiation of chondrogenesis.

## Graphical abstract

- Activation of Smad1/5 is critical in initiating chondrogenesis of multipotent progenitor cells (iMPCs) created from human induced pluripotent stem cells (iPSCs).
- iMPCs display rapidly decreased cartilage‐forming capacity with the increase of passage number.
- When subjected to pellet culture in the medium supplemented with TGFβ3 and BMP6, iMPCs can generate hyaline‐like cartilage with minimal chondrocytic hypertrophy phenotype.

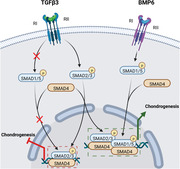

## 1 INTRODUCTION

Due to the limited self‐repairing capacity of hyaline cartilage, chondral defects often require osteochondral transplantation or cell implantation to support the regeneration process.^1^ Autologous chondrocyte implantation (ACI), which involves the isolation, expansion and implantation of autologous chondrocytes, is the most frequently employed cell‐based therapy in clinic. However, this therapy is limited by donor site morbidity, low donor tissue availability and loss of chondrocyte phenotype during in vitro expansion.^2^ Therefore, other cell types, such as nasal chondrocytes and mesenchymal stromal cells (MSCs), which are abundant and do not sacrifice the articular surface, have been tested for repairing cartilage injury. For example, MSCs from different sources have been shown to differentiate into chondrocyte‐like cells, indicated by the expression of representative chondrogenic genes and the capacity to produce the cartilage matrix. However, hypertrophic conversion often ensues, resulting in tissue fibrogenesis or ossification and apoptosis of the differentiated cells.^3^, ^4^ This hypertrophic conversion is not observed in native, healthy articular cartilage and chondrocytes. Many different methods for suppressing MSC hypertrophy have been tested, but long‐term stability assessments of MSC‐derived chondrocytes are lacking, leading many to propose that MSCs are innately programmed to undergo endochondral ossification with no capacity to generate hyaline cartilage.^5^, ^6^

Recently, induced pluripotent stem cells (iPSCs) have been investigated as a new cell source for generating articular cartilage.^7^, ^8^, ^9^, ^10^, ^11^ Especially, several studies have reported that iPSC‐derived chondrocytes expressed lower levels of hypertrophy markers,^12^, ^13^ implying their potential in regenerating hyaline cartilage. Currently, three different strategies are used to generate chondrocytes from iPSCs, including direct iPSC differentiation, sequential differentiation of iPSC embryoid bodies and formation of multipotent progenitor cells (iMPCs) from the iPSC and subsequent chondrogenesis.^14^ All of these methods require bone morphogenetic proteins (BMPs) in either the generation of progenitor cells or the differentiation stage. For example, recent studies have shown that transforming growth factor‐β (TGFβ), a potent chondro‐inductive factor for MSCs, is not sufficient to effectively induce iMPC chondrogenesis.^15^, ^16^, ^17^ However, the addition of BMP2 or four significantly enhanced TGFβ induced iMPC chondrogenesis. The mechanism underlying the different growth factor requirements between iMPCs and MSCs is not clear.

In this study, we first tested different growth factors alone or in combination in stimulating iMPC chondrogenesis, with a special focus on chondrocytic hypertrophy. The reparative capacity of iMPCs‐derived cartilage was tested in an osteochondral defect model created in rats. Human MSCs (hMSC) isolated from bone marrow, which represent the currently most tested stem cells for cartilage regeneration, were included in all studies as the control, enabling direct comparison between MSCs and iMPCs. In addition, a mechanistic study was conducted to understand why iMPCs and MSCs behave differently in responding to TGFβ. Specifically, we discovered that TGFβ resulted in the phosphorylation of both Smad2/3 and Smad1/5 and robust chondrogenesis in hMSCs, while TGFβ alone induced phosphorylation of Smad2/3 in iMPCs but not Smad1/5, which led to poor chondrogenesis. Supplementing BMP6 activated Smad1/5 in iMPC and significantly enhanced chondrogenesis, and the chondro‐promoting from BMP6 was abolished by the treatment of LDN193189, a BMP pathway inhibitor.

## 2 MATERIALS AND METHODS

### 2.1 hMSC isolation from human bone marrow

With the approval from Institutional Review Board (IRB) at the University of Pittsburgh and the University of Washington, hMSCs were isolated from the femoral heads as previously described.^18^ Cells were maintained in growth medium (GM, DMEM, Gibco, Grand Island, NY) with 1% (v/v) antibiotic‐antimycotic and 10% (v/v) fetal bovine serum (FBS) (Gibco). Fibroblast growth factor 2 (1.5 ng/ml) (PeproTech, Germany) was added to media during cell expansion. Cell culture media were changed twice a week. When reaching 70%–80% confluence, the culture was treated with trypsin‐0.25% (w/v)/ethylenediaminetetraacetic acid (EDTA) (ThermoFisher, Waltham, MA), and cells were passaged. hMSCs isolated from four de‐identified donors (70‐year‐old female [ID: 70F71614], 57‐year‐old male [ID: 57M032013], 51‐year‐old female [ID: 51F110718], 75‐year‐old male [ID: 75M032019]) were used. As completing the study required a large number of cells, we used the pooled hMSCs from these four donors in pellet culture for cartilage formation.

### 2.2 Generation of iMPCs from iPSCs

**FIGURE 1:**
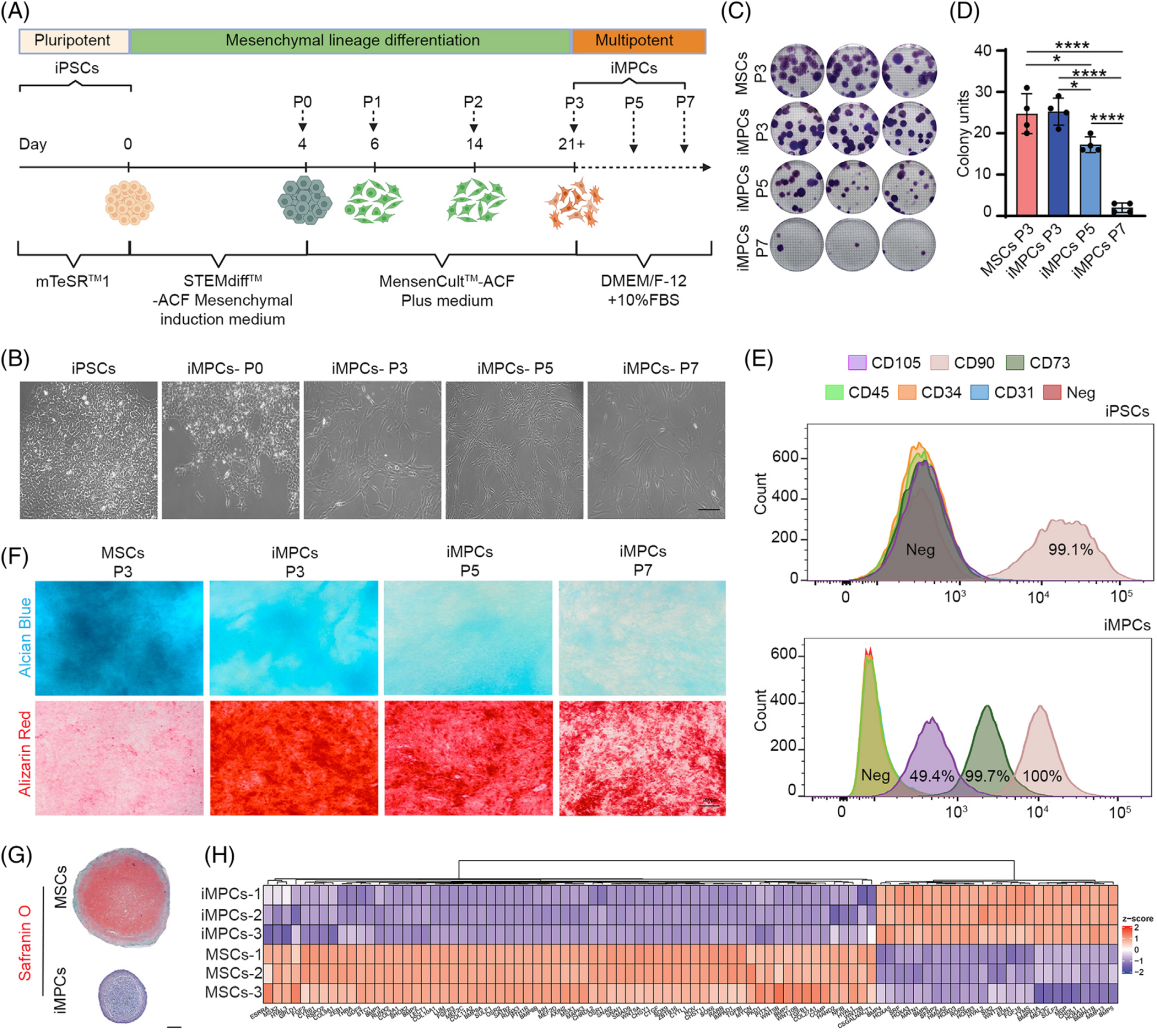
Generation and characterization of iMPCs derived from human iPSCs. (A) Schematic of mesenchymal lineage differentiation protocol for iPSCs. (B) Representative cell morphology of iPSCs and iMPCs at passages 0, 3, 5 and 7 (P0, 3, 5 and 7). Scale bar: 100 μm. (C and D) Colony‐forming unit (CFU) assay for human MSCs (P3) and iMPCs (P3, 5 and 7). The number of colonies from 100 cells was counted. *p < .05; ****p < .0001 (*N* = 4). One‐way ANOVA followed by Tukey's multiple comparisons test was carried out. (E) Flow cytometry analysis of surface marker expression in iPSCs and iMPCs. (F) Alcian Blue and Alizarin Red staining for MSCs and iMPCs (P3–7) after 21 days of culture in chondrogenic medium (basic medium [BM] supplemented with TGFβ3) and osteogenic medium. Scale bar: 200 μm. (G) Safranin O/Fast green staining for MSC (P3) and iMPC (P3) pellet cultures, which were maintained in chondrogenic medium (BM+TGFβ3) for 3 weeks. Scale bar: 200 μm. (H) Differentially expressed cartilage development‐associated transcripts in pellets formed by MSCs or iMPCs, after 21 days of culture in BM+TGFβ3 medium, were examined using RNA‐sequencing (*N* = 3).

Three human iPSC lines were tested. Line A‐iPSC, created from umbilical cord‐derived MSCs, was purchased from ALSTEM (Richmond, CA). W‐iPSC and C‐iPSC, respectively, derived from bone marrow MSCs (48 years old)^19^ and peripheral blood (79 years old) were gifts from Dr. Rocky Tuan's lab at the University of Pittsburgh and Cedars‐Sinai Medical Center. If not specified, results were collected from the studies using A‐iPSCs. However, the other two iPSC lines were used to validate key findings from A‐iPSCs. All media used in expanding and differentiating iPSCs were from STEMCELL Technologies (Vancouver, Canada). As described on the manufacturer's instructions, iPSCs were expanded on Vitronectin (STEMCELL Technologies), coated non‐tissue culture six‐well plates (Corning Life Sciences, Corning, NY) with mTeSR‐1 medium. The medium was refreshed every day. Once reaching optimal confluency, iPSCs were detached by ReLeSR (STEMCELL Technologies) or Gentle Cell Dissociation Reagent (STEMCELL Technologies). For passaging purposes, ReLeSR was used for detachment, and the iPSCs were re‐seeded at appropriate density with mTeSR‐1 medium. For generating iMPCs (Figure 1A), the iPSCs were first detached and re‐seeded with mTeSR‐1 medium supplemented with 10 mM ROCK inhibitor Y27632 (STEMCELL Technologies). At 80% confluency, iPSCs were treated with STEMdiff‐ACF Mesenchymal Induction Medium for 3 days, and then MesenCult‐ACF Plus Medium for 6 days. iMPCs were dissociated with ACF Enzymatic Dissociation Solution and ACF Enzyme Inhibition Solution (STEMCELL Technologies) and re‐seeded onto flasks coated with Animal Component‐Free Cell Attachment Substrate (STEMCELL Technologies). When reaching ∼80% confluence, iMPCs were expanded on generic tissue culture flasks in the growth medium (DMEM/F‐12 [Gibco, Grand Island, NY] supplemented with 10% FBS and 1% antibiotic‐antimycotic).

### 2.3 MSC/iMPC characterization

#### 2.3.1 Colony formation assay

One hundred‐mm tissue culture dishes (Thermo Fisher, Waltham, MA) were plated with 100 MSCs or iMPCs. After growing for 14 days in GM, phosphate‐buffered saline (PBS) was used to wash the dishes, and 0.5% Crystal Violet (Sigma‐Aldrich, St. Louis, MO) in methanol was administered and used to stain cells for 5–10 min at room temperature. PBS was used to rinse the culture following staining. The stained culture was imaged using a Nikon Eclipse E800 upright microscope (Nikon, Melville, NY). The number of colonies was manually counted.

#### 2.3.2 Flow cytometry

Trypsin was used to detach iMPCs. Following detachment and wash, iMPCs were incubated with different antibodies to examine the surface maker expression profile: FITC‐conjugated mouse anti‐human CD73, CD105, CD90, CD31, CD34 and CD45 (BD Biosciences, Franklin Lakes, NJ). All antibodies used for flow cytometry were conjugated with fluorescein isothiocyanate (FITC). One million cells were used for each antibody examination. Based on the suggestion from the manual, for the detection of CD31, CD34 and CD45, we took 20 μl antibody solution into 80 μl cell suspension. As for the CD73, CD90 and CD105, we took 5 μl antibody solution into 95 μl cell suspension. Propidium iodide (PI) was used for the selection of live cells at the concentration of 1:150. Flow cytometry (BD FACS AriaTM II cell sorter; BD Biosciences, NJ) was performed to assess the expression of MSC/iMPC surface epitopes. Gating strategy is shown in Figure S15.

#### 2.3.3 Trilineage differentiation

1. Osteogenesis

Osteogenic medium (OM: high‐glucose DMEM supplemented with 10% FBS, 1% antibiotic‐antimycotic, 0.1 μM dexamethasone [Sigma‐Aldrich], 10 mM β‐glycerophsphate [Sigma‐Aldrich], and 50 μg/ml ascorbate 2‐phosphate [Sigma‐Aldrich]) was used to culture MSCs/iMPCs in six‐well plates (20 000 cells/cm^2^). After 21 days, MSCs/iMPCs were fixed with paraformaldehyde and subjected to Alizarin Red staining (Rowley Biochemical, Danvers, MA).^20^ Procedure: For two‐dimensional (2D) samples, cells were fixed in 10% formalin for 15 min and washed copiously with tap water, then Alizarin Red solution 0.5%, pH = 4.2 (Cat# C‐206, Rowley Biochemical Institute) was added. The samples were incubated at room temperature for 5 min with gentle shaking. After aspiration of the unincorporated dye, the samples were washed with tap water. For three‐dimensional (3D) samples, the sections were deparaffinized in 60°C ovens for 1 h and with the wash of histoclear. Then, 100%, 95% and 70% ethanol were used to rehydrate the samples. Following incubation of Alizarin Red solution 0.5%, pH = 4.2 (Cat# C‐206, Rowley Biochemical Institute) for 30 s to 5 min with careful detection of the orange‐red colour under the microscope.

2. Chondrogenesis

Chondrogenic medium (CM: high‐glucose DMEM supplemented with 1% antibiotic‐antimycotic, 0.1 μM dexamethasone [Sigma‐Aldrich], 10 μg/ml ITS+ [ThermoFisher, Waltham, MA], 40μg/ml L‐proline [Sigma‐Aldrich], 50 μg/ml ascorbate 2‐phosphate [Sigma‐Aldrich], 10 ng/ml transforming growth factor β3 [TGFβ3] [Peprotech, Rocky Hill, NJ]) was used for MSCs/iMPCs seeded at a density of 20 000 cells/cm^2^ in six‐well plates. No FBS was supplemented. After 21 days, iMPCs were fixed with 4% paraformaldehyde and stained with Alcian Blue (EKI, Joliet, IL).^20^ Procedure: after 15 min of fixation, the samples were immersed with 3% acetic acid (v/v) in H_2_O for 15 min. Then, Alcian Blue solution pH = 1.0 (Cat# 1198, EK Industries, Joliet, IL) was added for 2 h of incubation at room temperature.

3. Adipogenesis

Adipogenic medium (AM: α‐MEM [Gibco, Grand Island, MY] supplemented with 10% FBS, 1% antibiotic‐antimycotic, 0.2 mM indomethacin [Sigma‐Aldrich], 0.1 μM dexamethasone [Sigma‐Aldrich], 1 μg/ml ITS+ [ThermoFisher, Waltham, MA]) was used for MSCs/iMPCs seeded at a density of 20 000 cells/cm^2^ in six‐well plates. After 21 days, iMPCs were fixed with 4% paraformaldehyde for 15 min and assessed with Oil Red staining (EKI, Joliet, IL)^21^ at room temperature for 2 h.

Due to the known donor‐to‐donor variation, trilineage differentiation of hMSCs from different patients was also separately performed. The results are shown in Figure S1.

#### 2.3.4 Pellet culture and chondrogenesis

Chondrogenic pellet culture was conducted by first resuspending 0.3 × 10^6^ MSCs or iMPCs in 200 μl basic chondrogenic medium (BM, high‐glucose DMEM supplemented with 1% antibiotic‐antimycotic, 0.1 μM dexamethasone, 40 μg/ml L‐proline, 10 μg/ml ITS+, 50 μg/ml ascorbate 2‐phosphate). Growth factors were added as indicated below. The concentrations of the growth factors were as follows:100 ng/ml bone morphogenetic protein (BMP)‐2 (PeproTech), 100 ng/ml bone morphogenetic protein (BMP)‐4 (PeproTech), 10 ng/ml transforming growth factor‐beta (TGFβ3, PeproTech, Germany) combined with 100 ng/ml bone morphogenetic protein (BMP)‐6 (PeproTech). Cell suspension was then plated in 96‐conical well plates and subjected to centrifugation at 300 × *g* for 10 min to form pellets. Medium was replaced every 2 days, and pellets were harvested after 7, 14 and 21 days for further analysis.

To examine the function of activating Smad1/5 during the chondrogenic differentiation in iMPCs, LDN‐193189^22^ (LDN; Selleck Chemicals, Houston, TX), a Smad1/5 pathway inhibitor was added at 0.5 μM to the basic chondrogenic medium supplemented with TGFβ3+BMP6. Dimethyl sulfoxide (DMSO) was used as vehicle control. Phosphorylated Smad1/5 (pSmad1/5) levels were assessed by Western blot at day 7. In this study, the different time periods of using LDN‐193189 during the total 21‐day chondrogenic process was done to test the function of activating Smad1/5 in the chondrogenesis of iMPCs.

### 2.4 Total RNA isolation and quantitative reverse transcription PCR

Cell total RNA was extracted using QIAzol from the RNeasy Plus Mini Kit (Qiagen, Germantown, MD, USA). The enzyme used for reverse transcription was SuperScript IV VILO (Invitrogen, Waltham, MA) according to the manufacturer's protocol. The extracted total RNA was prepared for bulk RNA‐sequencing (RNA‐seq) and quantitative reverse transcription PCR (RT‐qPCR). RT‐qPCR was conducted using SYBR Green Reaction Mix (Applied Biosystems, Foster City, CA, USA) with the QuantStudio 3 RT‐qPCR system (Applied Biosystems, Foster City, CA). The comparative Ct (2‐ΔΔ*CT*) method was used to calculate the relative gene expression. Ribosomal protein L13A (RPL‐13A) or glyceraldehyde 3‐phosphate dehydrogenase (GAPDH) was used as the endogenous control gene. The sequences of primers are listed in Table S1.

### 2.5 GAG assay

Sulfated glycosaminoglycan (GAG) content was quantified using a GAG assay. Samples were washed with HBSS, homogenized and then digested by papain (125 μg/ml papain, 50 mM sodium phosphate buffer, 2 mM *N*‐acetyl cysteine [Sigma‐Aldrich], pH = 6.5). The suspension was centrifuged at 12 000 × *g* for 5 min, and the supernatant was collected for GAG assay (Blyscan, Biocolor, UK). DNA content was determined with the Picogreen dsDNA assay (Molecular Probes, Tarrytown, NY) for GAG normalization.

### 2.6 Histology and immunohistochemistry

Pellets were fixed using 10% buffered formalin (Fisher Chemical, Hampton, NH) and incubated overnight at 4 C, then dehydrated in ethanol and xylene and embedded in paraffin (Fisher). Paraffin blocks were sectioned into 6‐μm thick sections using a Leica microtome (Model RM 2255). Slides were stained using safranin‐O (0.5% in 1% acetic acid)/fast green (0.04% in 0.2% acetic acid) or alizarin red‐S (0.5% in water) as previously described.^23^ Imagining was conducted using a Nikon Eclipse E800 upright microscope.

For Immunohistochemistry, the formalin‐fixed paraffin‐embedded sections first underwent antigen retrieval based on different antibodies. Slides were then blocked with 10% goat serum (Abcam, Cambridge, MA) in PBS for 1 h, incubated at 4°C overnight with the primary antibody against collagen type II (COL2, clone 4c11, MP Biomedicals/Quartett, 1:1000 dilution), collagen type I (COL1, Abcam, Cambridge, MA, 1:100), collagen‐type X (COL10, Invitrogen, Carlsbad, CA, 10 μg/ml), IHH (Abcam, Cambridge, MA, 1:50 dilution), then incubated with a biotinylated anti‐mouse/rabbit immunoglobulin G (IgG) secondary antibody for 1 h, with signal detection via DAB substrate kit (Abcam). Haematoxylin was used for counterstaining (Vector Laboratories, Burlingame, CA). To image the stained sections, an Olympus IX81 inverted microscope (Olympus, Shinjuku City, Tokyo, Japan) was used. Antibody information is included in Table S2.

### 2.7 Western blot

Total protein extracts, without prior pepsin digestion, were prepared using RIPA buffer solution (Sigma‐Aldrich) supplemented with protease and phosphatase inhibitor cocktail (1% [v/v], ThermoFisher). Total protein concentrations were determined using the BCA protein assay kit (Thermo Scientific BCA Protein Assay Kit). 4%–12% Bis‐Tris polyacrylamide gels (Invitrogen, NP0326BOX) were used for loading protein samples and subjected to electrophoresis (200 V, 50 min). Then semi‐dry transfer or wet transfer was used for transferring the protein blot from the gel to the membrane. Polyvinylidene fluoride membranes with low fluorescence background (Millipore, Billerica, MA, USA) were used in a wet transfer manner, and iBlot‐2 Transfer Stacks were used in a semi‐dry manner by iBlot 2 Dry Blotting System (Invitrogen). Membranes were blocked in 3% milk in Tris‐buffered saline with Tween‐20 (TBS‐T, 0.1% Tween‐20) for 1 h, and 1% milk/TBS‐T was used to incubate membranes overnight at 4°C with primary antibodies. Horseradish peroxidase (HRP)‐conjugated secondary antibodies (Thermo Scientific) were used to perform immunodetection followed by Super Signal West Dura Extended Duration Substrate (Thermo Scientific Pierce Protein Biology), and then imaged with ChemiDoc Touch Imaging system (Bio‐Rad, Hercules, CA). Antibody information is provided in Table S2.

### 2.8 In vivo subcutaneous implantation in mice

Animal study was approved by the University of Pittsburgh Institutional Animal Care and Use Committee (IACUC). Severe combined immunodeficiency (SCID) male mice (Jackson Laboratory, Bar Harbor, Maine) at 8–10 weeks old were used. Subcutaneous implantation was performed on the dorsal region of the mouse. Mice were anaesthetized with 2% isoflurane carried by oxygen, and the skin was shaved and sterilized over the implantation area using standard sterile techniques. An ∼6 mm skin incision was created, and different groups of chondrogenic pellets inserted into the skin incision. In this study, three groups for two time points were used. (a) MSC‐derived chondrogenic pellets were cultured in the basic chondrogenic differentiation medium supplemented with TGFβ3. (b) MSC‐derived chondrogenic pellets were cultured in the basic chondrogenic differentiation medium supplemented with TGFβ3+BMP6. (c) iMPC‐derived chondrogenic pellets were cultured in the basic chondrogenic differentiation medium supplemented with TGFβ3+BMP6. All the pellets from the three groups (three pellets each group) were cultured for 21 days before implantation (four mice per group). The incisions were closed with sutures post implantation. After 14 and 21 days, the mice were sacrificed, and chondrogenic pellets were collected for micro‐computed tomography (micro‐CT) analysis and histology.

### 2.9 Micro‐CT analysis

The chondrogenic pellets that were collected from mice were fixed in 10% buffered formalin (Fisher Chemical, Hampton, NH) for micro‐CT analysis of calcification. Micro‐CT (μCT, Scanco Medical vivaCT 40, Brüttisellen, Switzerland) scans were performed at 45 kVp, 88 μA, 300 ms integration time and acquired at an isotropic voxel size of 35 μm. The raw 2D μCT image slides were used to reconstruct the 3D specimens. Bone volume (BV) and bone mineral density (BMD) were quantified using the Scanco evaluation software.

### 2.10 Cartilage repair in a rat osteochondral defect model

For the implantation in the osteochondral defects in the knee joint, male RNU nude rats were used, which were purchased from Charles River Laboratories (Wilmington, MA). An osteochondral defect (2 mm in diameter and 2 mm in depth) was made in the trochlear groove of the femur by a Stoelting Cordless Micro Drill (Fisher Scientific). MSC/iMPC‐derived chondrogenic pellets were inserted into defect sites. In this study, there were four groups and three rats in each group as follows: (a) MSC‐derived chondrogenic pellets cultured in the basic chondrogenic differentiation medium supplemented with TGFβ3 for 21 days; (b) iMPC‐derived chondrogenic pellets cultured in the basic chondrogenic differentiation medium supplemented with TGFβ3+BMP6 for 21 days; (c) sham group (without creating osteochondral defect); and (d) no implantation group (osteochondral defects were not filled with implants). After implantation, the patella was repositioned to its original anatomical location, the subcutaneous layer and skin were closed with 4‐0 suture. In the following 3 days, the animals were treated with Buprenex (subcutaneous [SQ] injection, 0.1 mg/kg) and enrofloxacin (SQ injection, 5 mg/kg). Four rats were used in each group (*N* = 4). After 8 weeks, the rats were sacrificed, and the knee joints were harvested in 10% buffered formalin (Fisher Chemical, Hampton, NH) for 24 h, then underwent micro‐CT analysis. Samples were decalcified by Immunocal Decal Solution (StatLab, McKinney, TX) for 4 weeks. After that, samples underwent sequential dehydration in different concentrations of ethanol (from 25%, 50%, 75% to 100%) for 2 h each, cleared in xylene for 1 h and embedded in paraffin. Paraffin blocks were sectioned into 6 μm thick sections.

### 2.11 Bulk RNA‐seq data analysis

For bulk RNA‐seq experiments, cell pellets made by iMPCs or BMSCs were induced in the traditional chondrogenic medium (containing TGFβ3 only) for 21 days, and then lysed in QIAzol from the RNeasy Plus Mini Kit (Qiagen, Germantown, MD, USA) for RNA extraction. The extracted total RNA was prepared for bulk RNA‐seq. The sequencing is done on Illumina NovaSeq 6000 platform. It is 101PE reads. The library preparation was using KAPA hyper mRNA kit. Quality control was first performed on the bulk RNA‐seq data by tool FastQC (refer to https://www.bioinformatics.babraham.ac.uk/projects/fastqc/). Adapter sequences or low‐quality reads were filtered out by tool Trimmomatic.^24^ The processed reads were then aligned to human reference genome hg38 by STAR aligner^25^ for gene quantification. All the tools were run by default parameter settings. Raw read count data were used to perform differential expression analysis. R package ‘DESeq’^26^ was applied to perform the differential test. Differentially expressed genes (DEGs) were defined by FDR = 5% and fold‐change higher than 1.5‐fold. These genes were further applied to ingenuity pathway analysis (IPA, refer to https://digitalinsights.qiagen.com/products‐overview/discovery‐insights‐portfolio/analysis‐and‐visualization/qiagen‐ipa/) to detect the enriched pathways. FDR = 5% was used to define significant pathways. Genes involved in the selected pathways were visualized by heatmap drawn by R package ‘ComplexHeatmap’^27^ and ‘ggplot’.^28^ All the statistical analyses were performed by R programming. RNA‐sequencing data were uploaded to the Gene Expression Omnibus with accession ID: GSE197172.

### 2.12 Statistical analysis

All the data were presented as mean ± SD. GraphPad Prism 9 (GraphPad, San Diego, CA) was used for statistical analysis. In general, analysis of one‐way analysis variance (ANOVA) was used to analyze results among multiple groups, and mean differences between the two groups were assessed with unpaired *t*‐test. The type of analysis conducted has been specified in each figure legend. *p* < .05 was considered statistically significant.

## 3 RESULTS

### 3.1 Rapid loss of differentiation capacity of iMPCs with increasing passage number

The process of creating iMPCs from iPSCs is shown in Figure 1A. In brief, iPSC cultures were exposed to the sequential treatments of different media without undergoing an intermediate embryoid body (EB) formation process. As the application of iMPCs in cartilage tissue engineering may require a large number of cells, we expanded iMPCs up to passage 7 and examined the differentiation potential of iMPCs with increasing passage numbers.

We first noted a consistent progression of cell shape from round (iPSCs) to polygonal (iMPCs‐P0) to spindle‐like (iMPCs‐P3‐7). Representative cell morphology at increasing passage numbers is shown in Figure 1B. Next, we examined the stemness of iMPCs at passages 3, 5 and 7 with traditional methods used to characterize hMSCs, including CFU‐F assay, cell surface marker expression and trilineage differentiation. Human bone marrow‐derived MSCs at passage 3 (P3) were employed as the control. All studies were repeated using two iPSC lines (A‐ and W‐iPSCs) and four MSC lines isolated from different donors (Figure S1).

First, we assessed the colony‐forming capacity of iMPCs with increasing passage numbers. One hundred iMPCs at P3 could form around 24 colonies, which was similar to P3 MSCs (Figure 1C,D). However, at P7, 100 iMPCs only formed approximately four colonies, demonstrating the detrimental influence of in vitro expansion on iMPCs’ stemness.

Second, we examined the surface marker profile of the iMPCs. As shown in Figure 1E, we found that the parent iPSCs were positive only for CD90. In contrast, P3 iMPCs possessed a similar surface marker profile to P3 MSCs (Figure S2), such as high expression levels of CD90 (100%) and CD73 (99.7%), but not CD31, CD34 and CD45. Of note, CD105 was represented in 49.4% of iMPCs.

Third, we tested iMPC potential at P3 in 2D culture using the well‐established differentiation protocols. In comparison to P3 MSCs, P3 iMPCs displayed lower chondrogenic potential but higher osteogenic potential (Figure 1F and Figure S1). Interestingly, the conventional adipogenic medium for MSCs was not able to induce adipogenesis in iMPC culture. However, using a different type of medium^29^ in which specific components, such as SB431542, epidermal growth factor and hydrocortisone, were provided, iMPCs could also undergo robust adipogenesis (Figure S3). These results indicated that iMPCs and MSCs might need different media to achieve a high level of differentiation. In addition, the qualitative histological data demonstrated that the differentiation capacity of iMPCs decreased from P3 to P7 (Figure 1F). Interestingly, the ratio of CD105‐positive cells decreased to 37.8% and 41.5% in P5 and P7 iMPCs (Figure S4).

Because a major focus of this work is to generate cartilage from iMPCs for chondral repair, we further assessed iMPC chondrogenesis in 3D pellet culture. Consistent with the result from 2D culture, the conventional chondrogenic medium only supplemented with TGFβ3 could not induce robust chondrogenesis of iMPC pellets (Figure 1G). Results from RNA‐seq (Tables S3 and S4) revealed that fewer cartilage development‐associated genes were upregulated in chondro‐induced iMPC cultures than MSC cultures (Figure 1H).

In summary, iMPCs rapidly lose differentiation capacity with increasing passage numbers. In addition, conventional TGFβ‐containing chondrogenic medium induces minimal to mild iMPC chondrogenesis in either 2D or 3D culture.

### 3.2 Optimization of conditions to create hyaline cartilage‐like tissues from iMPCs

**FIGURE 2:**
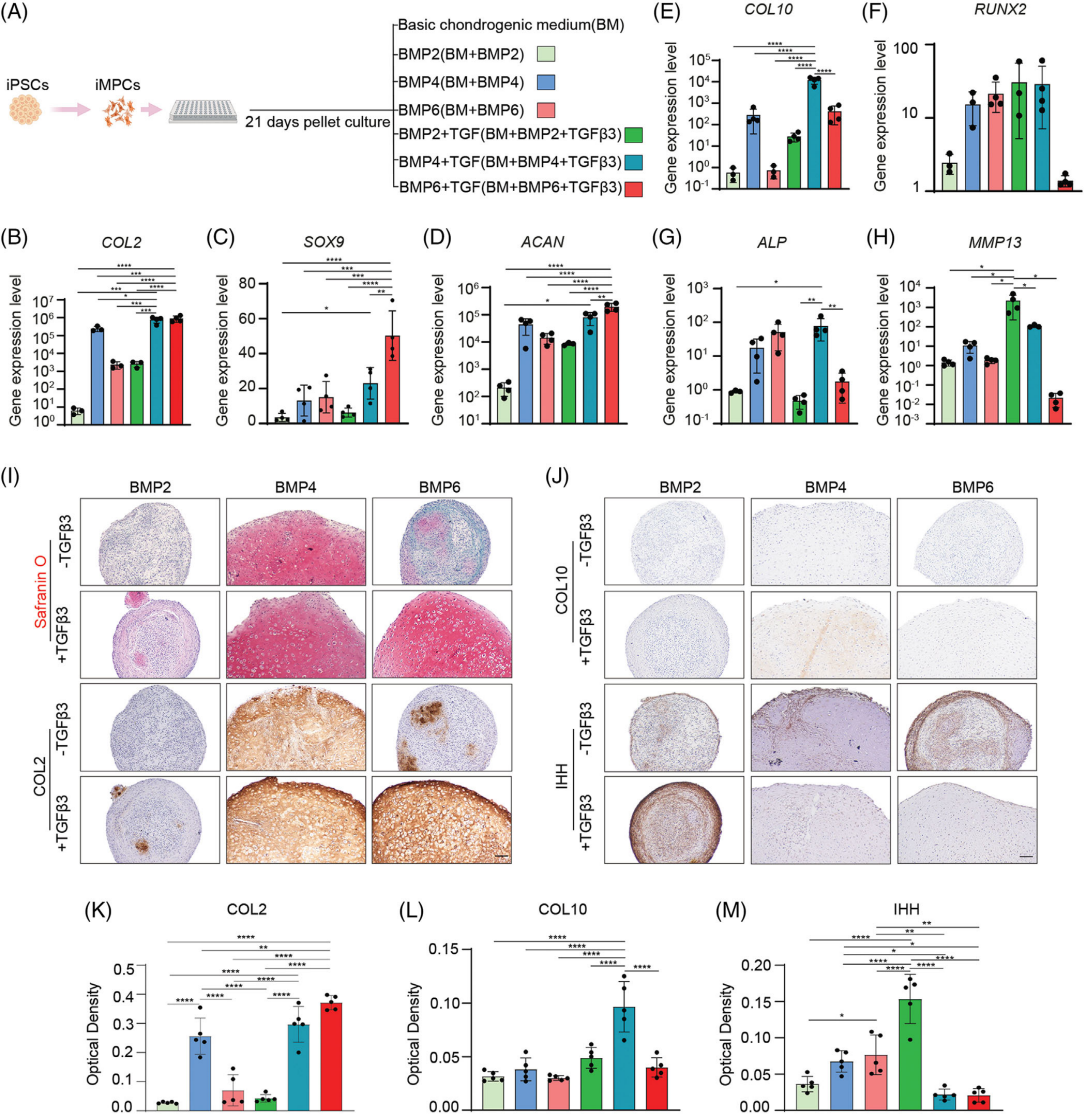
Chondrogenesis of human iMPCs with different media. (A) Schematic of procedures and corresponding experimental groups of inducing differentiation from iPSCs to cartilage tissue. Basic medium (BM) supplemented with different BMPs, with or without the addition of TGFβ3, was used to induce chondrogenesis. (B–H) Relative expression levels of chondrogenesis‐related markers (*COL2*, *SOX9* and *ACAN*) and hypertrophy/osteogenesis‐related markers (*COL10*, *RUNX2*, *ALP* and *MMP13*). Data were normalized to that in BM group (set as 1). BM is not shown in the figure. (*N* = 4). One‐way ANOVA followed by Tukey's multiple comparisons test was carried out. (I) Safranin O staining and COL2 immunohistochemical staining (IHC), and (J) COL10 and IHH IHC for iMPCs after 21 days of pellet culture in different media. Scale bar: 100 μm. (K–M) Quantitative optical density of IHC staining for COL2, COL10 and IHH (*N* = 5). One‐way ANOVA followed by Tukey's multiple comparisons test was carried out. *p < .05; **p < .01; ***p < .001; ****p < .0001

To define a condition that could result in the formation of hyaline cartilage from iMPCs, we maintained iMPC pellet cultures in the basic chondrogenic medium (BM) containing BMP 2, 4 and 6, with or without the supplementation of TGFβ3 (Figure 2A).

As shown in Figure 2B–D, stimulating iMPC pellet cultures with BMP2 and BMP6 alone failed to induce a high level of chondrogenesis, while BMP4 alone resulted in a robust increase in chondrogenic gene expression, including collagen type II (*COL2*) and Aggrecan (*ACAN*) (Figure S5). However, when compared to BMP2 and BMP6, BMP4 also induced strong expression of molecules associated with hypertrophy, including collagen type X (*COL10)* and matrix metallopeptidase 13 (*MMP13)* (Figure 2E,H). BMP6 promoted the highest expression of Runt‐related transcription factor 2 (*RUNX2*) and alkaline phosphatase (*ALP*) in three tested BMPs (Figure 2F,G).

The addition of TGFβ3 slightly enhanced BMP4‐stimulated iMPC chondrogenesis, such as promoting higher *COL2* expression (approximately two times higher) (Figure 2B), which however was at the expense of a 10 times increase in *COL10* expression (Figure 2E). Of note, the addition of TGFβ3 to BMP2‐induced cultures also promoted iMPC chondrogenesis but significantly increased *MMP13* expression (Figure 2H).

BMP6 alone induced very low expression of chondrogenic genes; however, the co‐treatment with BMP6 and TGFβ3 induced high levels of chondrogenic gene expression, which were statistically greater than those stimulated with BMP4 (Figure 2B–D). Interestingly, expression levels of hypertrophic and osteogenic markers, including *RUNX2*, alkaline phosphatase (*ALP)* and *MMP‐13*, were remarkably lower in BMP6+TGFβ3 co‐treatment group, when compared to the BMP4 group (Figure 2F–H). These patterns in gene expression among different groups were further supported by histological and immunohistochemical (IHC) results (Figure 2I–M). For example, the highest levels of Safranin O staining (Figure 2I) and COL2 staining (Figure 2I,K) were observed in iMPC cultures stimulated with BMP4, BMP4+TGFβ3 or BMP6+TGFβ3. However, the BMP4+TGFβ3‐stimulated iMPC cultures also displayed the highest level of COL10 staining (Figure 2J,L). Indian hedgehog (IHH), a regulator of hypertrophy, was observed in cultures stimulated with BMP alone, where IHH was qualitatively highest in the BMP4 and BMP6 groups (Figure 2J,M). Interestingly, the addition of TGFβ3 to BMP6‐stimulated cultures (BMP6+TGFβ3 group) significantly reduced IHH production while enhancing IHH production in the BMP2‐stimulated group.

In addition to the results shown above, we also examined other conditions and defined that the BM supplemented with both BMP6 and TGFβ3 promoted the highest iMPC chondrogenesis (Figure S6). Therefore, this chondro‐induction condition was used in the subsequent studies.

### 3.3 Comparison of chondrogenesis between primary MSCs and iMPCs

As primary MSCs represent the most tested stem cell source for cartilage repair, we compared iMPCs‐derived cartilage to that generated from primary MSCs. The experimental groups for this included MSC and iMPC cultures grown in BM only (BM group), or BM supplemented with TGFβ3 (TGF group), BMP6 (BMP group) or TGFβ3+BMP6 (T+B group).

**FIGURE 3:**
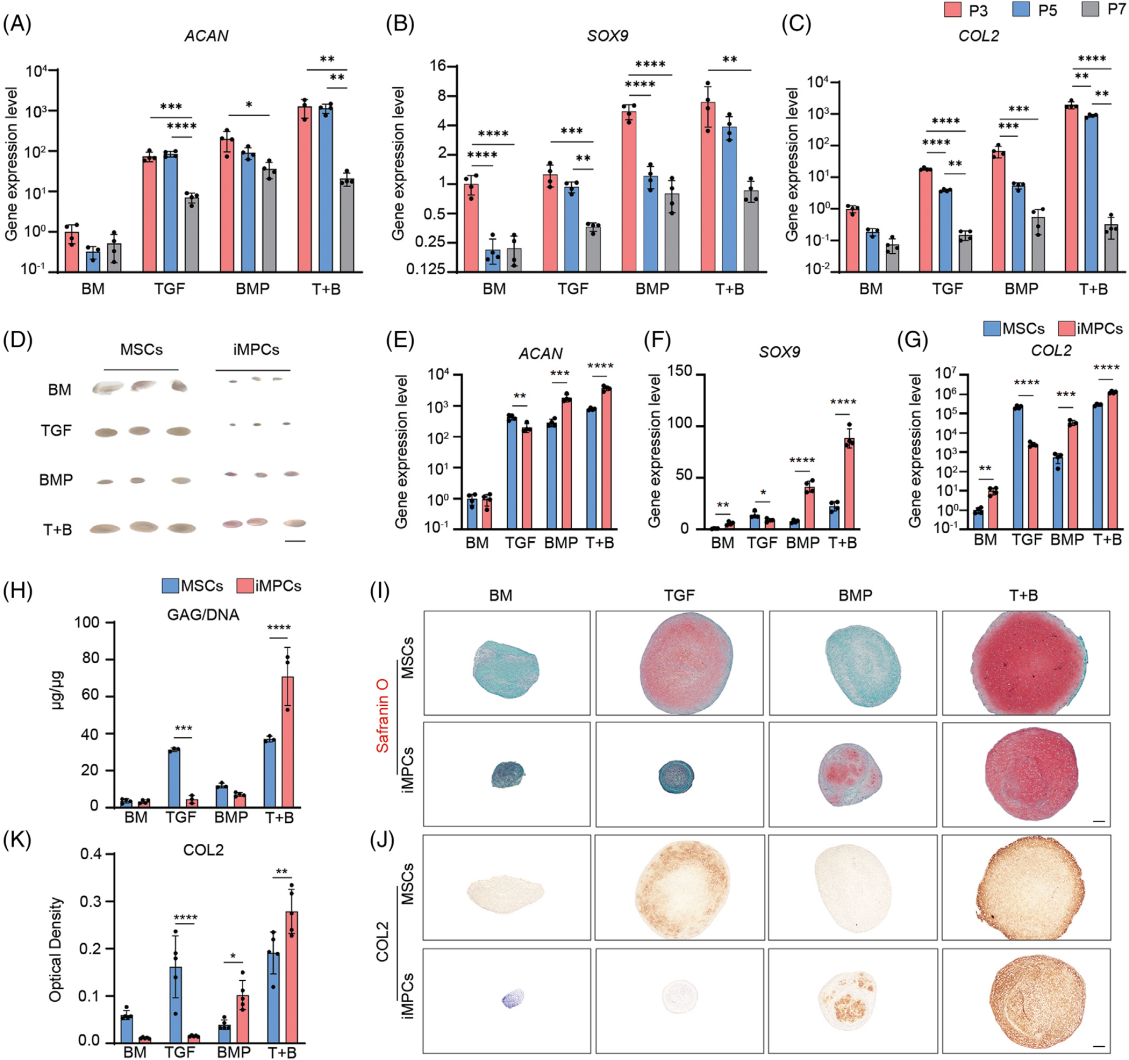
Characterization of cartilage derived from MSCs and iMPCs under different chondro‐inductive conditions. MSCs or iMPCs (derived from A‐iPSCs) were formed into pellets and then subjected to chondrogenic culture in basic chondrogenic medium (BM group), BM with TGFβ3 (TGF group), BM with BMP6 (BMP group) or BM with both TGFβ3 and BMP6 (T+B group) for 21 days. (A–C) Expression levels of *ACAN, SOX9* and *COL2* in cartilage tissues created by P3, P5 or P7 iMPCs. Data were normalized to P3 in the BM group (set as 1) (*N* = 4). One‐way ANOVA followed by Tukey's multiple comparisons test was carried out. (D) Macro‐appearance of P3 MSC‐ and P3 iMPC‐derived cartilage tissues. Scale bar: 5 mm. (E–G) Expression levels of *ACAN, SOX9* and *COL2* in cartilage tissues created by MSCs and iMPCs. Data were normalized to that in MSC/BM group (set as 1) (*N* = 4). Unpaired *t*‐test was carried out. (H) Measurements of glycosaminoglycans (GAG)/DNA (*N* = 3). Unpaired *t*‐test was carried out. (I) Safranin O staining and (J) COL2 immunohistochemical (IHC) staining. Scale bar: 200 μm. (K) Optical density of COL2 IHC staining (*N* = 5). Unpaired *t*‐test was carried out. *p < .05; **p < .01; ***p < .001; ****p < .0001

It was not clear at which passage iMPCs displayed the highest chondrogenesis when the newly optimized medium (BM supplemented with TGFβ3+BMP6) was applied. We thus first re‐examined the chondrogenic potential of P3, P5 and P7 iMPCs. As shown in Figure 3A–C and Figure S7, among four tested conditions, the highest chondrogenesis of iMPCs was again observed in T+B group. Moreover, pellet cultures of P3 iMPCs displayed the highest expression levels of chondrogenic genes (Figure 3A–C) and the highest level of Safranin O staining (Figure S8) when compared to P5 and P7 iMPCs under all experimental conditions. Therefore, the use of newly defined medium did not change the conclusion that chondrogenic potential of iMPCs reduced with the increase of passage numbers. P3 iMPCs were thus used in all the following studies, and primary hMSCs at the same passage (P3 MSCs) were used as the control.

After 21 days of differentiation, P3 MSC culture in TGF and T+B groups, and iMPCs culture in T+B group, were able to generate cartilaginous tissues (Figure 3D), which was supported by the high expression of chondrogenic genes (Figure 3E–G) and robust deposition of GAG (Figure 3H,I) and COL2 (Figure 3J,K). Interestingly, the biochemical assay showed that iMPCs generated more cartilage matrix than MSCs when BM supplemented with TGFβ3+BMP6 was used. For instance, chondrogenic iMPCs deposit ∼80% more GAG than MSCs (Figure 3H).

### 3.4 Comparison of chondrocyte hypertrophy in primary MSCs and iMPCs

**FIGURE 4:**
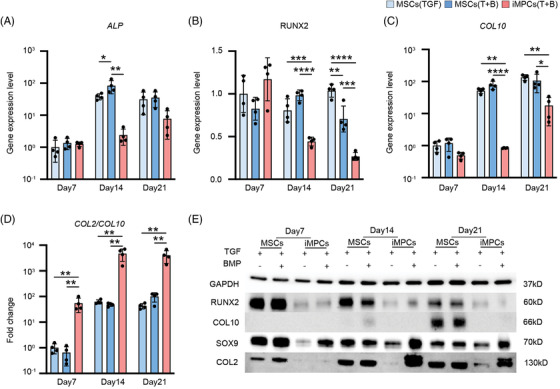
Characterization of hypertrophic phenotype in chondrocytes derived from MSCs and iMPCs under different chondro‐inductive conditions. (A–D) Relative expression levels of *ALP, COL10* and *MMP13*, and *COL2/COL10* ratio in cartilage tissues created by MSCs and iMPCs were examined by qRT‐PCR at different time points. Basic chondrogenic medium (BM) supplemented with TGFβ3 (TGF) or TGFβ3+BMP6 (T+B) was used. Data were normalized to that in MSCs (TGF) group (set as 1) (*N* = 4). One‐way ANOVA followed by Tukey's multiple comparisons test was carried out. (E) The relative protein levels of SOX9, COL2, RUNX2 and COL10 at different time points were examined by Western blot (*N* = 3). *p < .05; **p < .01; ***p < .001; ****p < .0001

Next, we assessed the hypertrophy level in cartilage generated by P3 MSCs and iMPCs. First, we examined the expression of hypertrophy genes under different chondrogenic conditions at different time points. The results showed that under both the conventional induction scheme (TGF β3 as the single chondro‐induction growth factor) and the optimized scheme (TGFβ3+BMP6), MSCs‐derived tissue always displayed higher expression of hypertrophy and osteogenesis‐related genes, such as *ALP, RUNX2* and *COL10*, than those formed by iMPCs (Figure 4A–C). In addition, the level of *COL2/COL10* gene expression ratio in iMPCs (T+B) group was ∼50 times higher than the MSC (TGF or T+B) groups on day 14 or 21(Figure 4D). Results from Western blot analysis further supported these findings (Figure 4E). On day 21, high levels of SOX9 and COL2 proteins were observed in MSC and iMPC (T+B) groups. However, the cartilage in MSC groups expressed high levels of RUNX2 and COL10, which was not seen in iMPC groups in all tested time points. Interestingly, on day 7, COL2 was detected only in the MSC group, suggesting that the chondrogenesis in iMPCs was slower than MSCs at the beginning.

**FIGURE 5:**
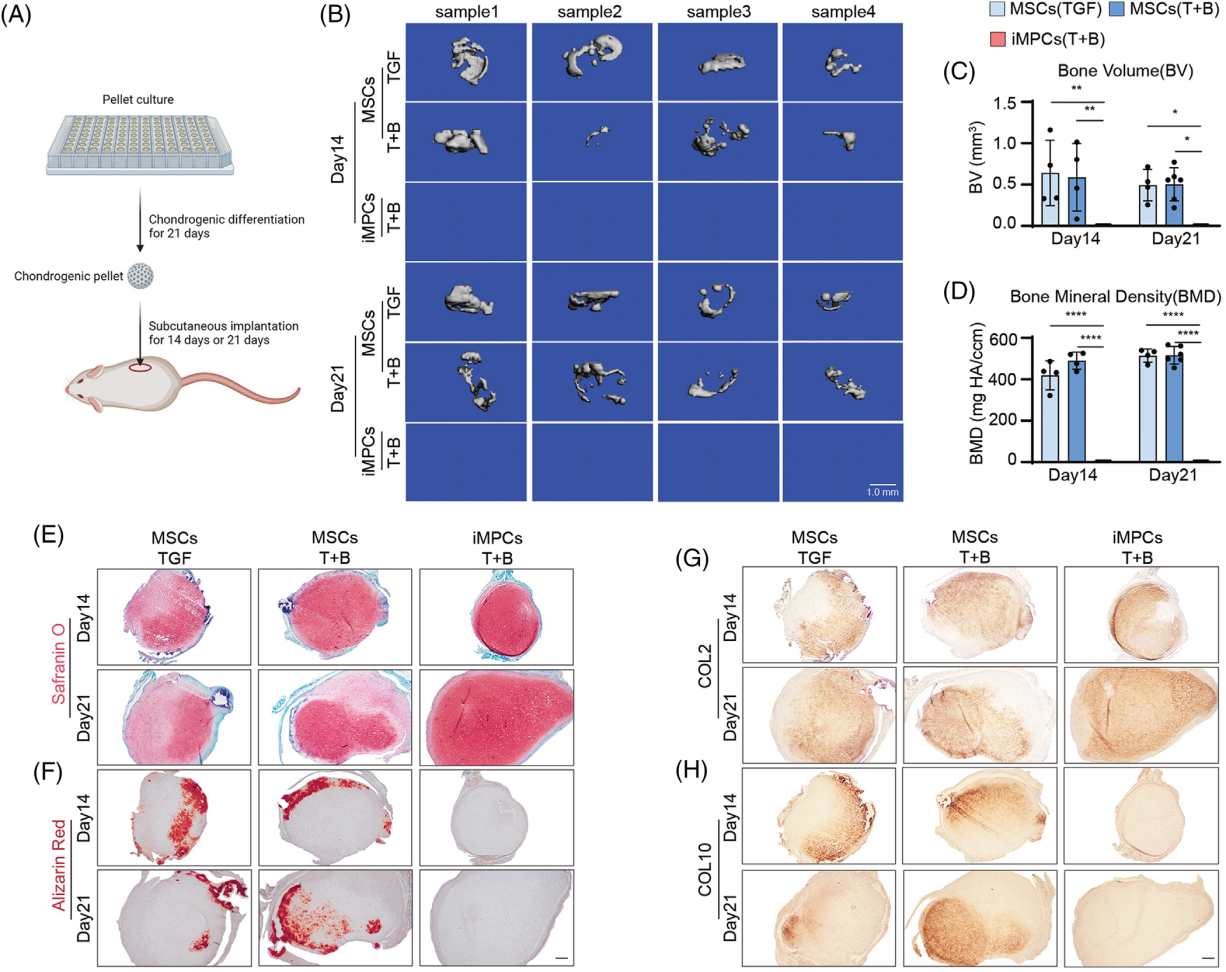
Assessment of chondrocytic hypertrophy level in cartilage tissues using subcutaneous implantation model. (A) Schematic of assessing the osteogenic potential of MSC or iMPC‐derived cartilage tissues in murine model. (B) micro‐CT imaging to show the formation of mineralized tissues in MSC groups (*N* = 4). (C–D) Bone volume and bone mineral density on days 14 and 21 post implantation were measured from micro‐CT analysis (*N* = 4). One‐way ANOVA followed by Tukey's multiple comparisons test was carried out. (E) Safranin O staining, (F) Alizarin Red staining, (G) COL2 IHC, and (H) COL10 IHC for retrieved implants. *N* = 3. Scale bar: 200 μm. *p < .05; **p < .01; ***p < .001; ****p < .0001

One of the consequences of chondrocyte hypertrophy is the transition to osteogenesis upon exposure to appropriate environmental cues. Therefore, we used subcutaneous implantation in a murine model to assess the hypertrophy level of cartilaginous neotissues generated by MSCs or iMPCs (Figure 5A). The samples retrieved on day 14 or 21 post‐implantation were first subjected to micro‐CT examination. The formation of mineralized tissues was observed in MSC groups as early as 14 days, as revealed by micro‐CT assay (Figure 5B–D). In contrast, no bone tissue was found in iMPC groups in 21 days. Histology and IHC of implants further revealed that constructs comprised of MSCs were undergoing robust ossification, as indicated by Alizarin Red staining (Figure 5F) and COL10 IHC (Figure 5H and Figure S9). Of note, cartilage tissue‐derived iMPCs maintained high levels of GAG and COL2 during implantation (Figure 5E,G and Figure S9). The immunofluorescence analysis detecting human nuclear antigen showed that the harvested pellets mainly comprised human cells (Figure S10).

In summary, the cartilage generated from iMPCs, induced by BM supplemented with TGF β3+BMP6, underwent minimal hypertrophic transition when compared to those from MSCs. The results implied iMPCs might represent a better cell source than MSCs in repairing hyaline cartilage.

### 3.5 Repair of osteochondral defects in rats

**FIGURE 6:**
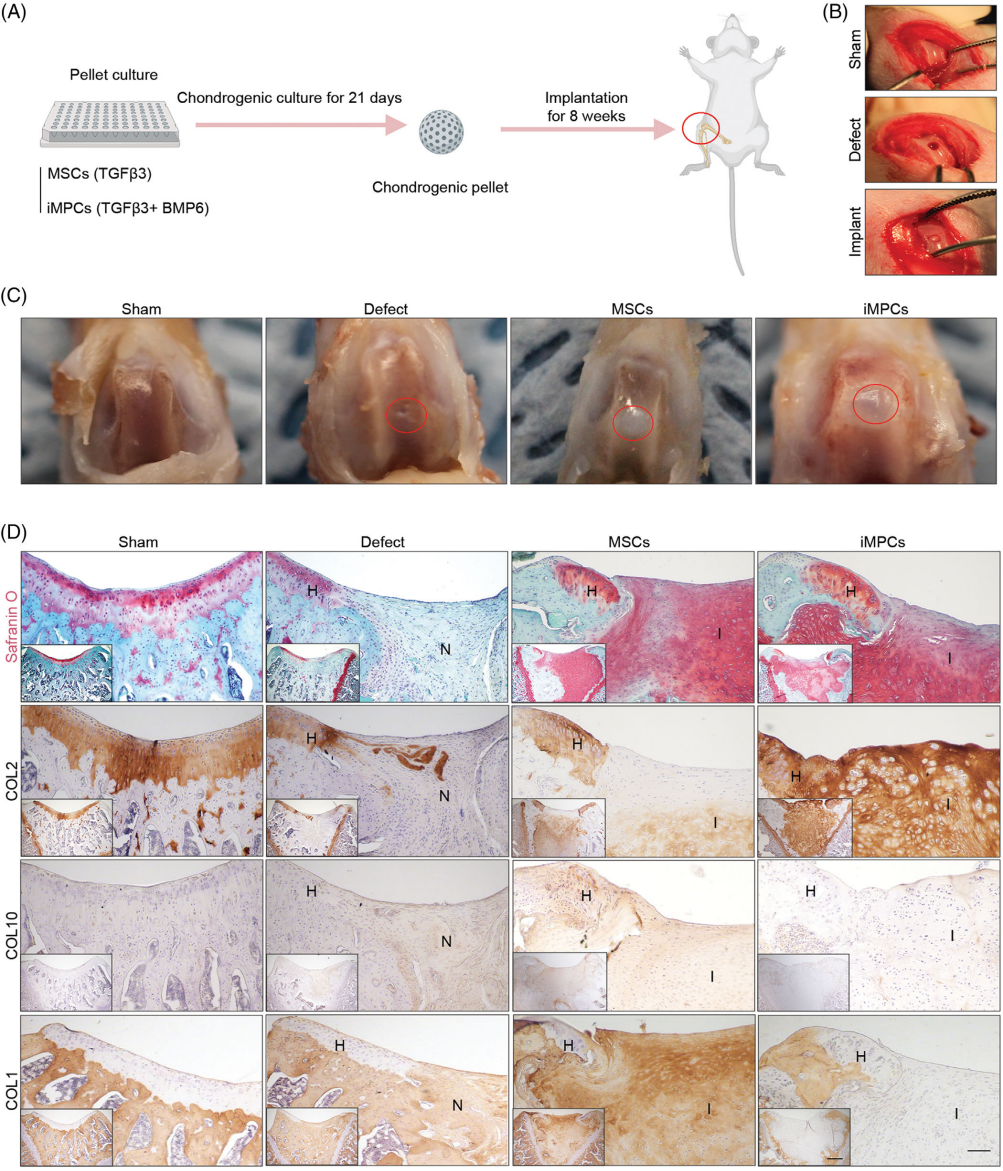
Repair of osteochondral defects in rat. (A) MSC‐ or iMPC‐derived cartilage tissues, chondro‐induced by BM with TGFβ3 or BM with TGFβ3+BMP6, respectively, were implanted into surgically created osteochondral defects in rats, which had a cylindrical shape (2 mm diameter × 2 mm depth). After 8 weeks, animals were euthanized, and knee joints were collected for analysis. (B) The process of surgery. (C) Macro‐appearance of the defects in four groups 8 weeks post surgery. (D) Safranin O staining, and COL2, 10, and 1 IHC for assessing the cartilaginous phenotype at the defect site. H = host tissue; I = implant; N = newly formed tissue that filled the defects. *N* = 3. Scale bar: 100 μm

To examine the reparative capacity of iMPCs, iMPC‐ or MSC‐derived cartilage pellets were implanted into surgically created osteochondral defects in the patellofemoral groove of rat knee joints (Figure 6A,B). After 8 weeks, regeneration of cartilage was assessed. As shown in Figure 6C and Figure S11, both implantation of MSC‐ and iMPC‐derived cartilages (MSCs and iMPCs groups) filled the defect, while untreated injury (Defect group) remained partially filled. Histology and IHC were employed to assess the quality of implants generated by MSCs or iMPCs at the defect site. Imaging of representative regions of typical samples at low and high magnifications was provided (Figure 6D). Safranin O staining revealed robust GAG deposition in both MSCs and iMPCs groups. Interestingly, there was significantly less deposition of COL2 in the MSC group in comparison to the iMPC group. Very limited GAG and COL2 deposition was noticed in the Defect group. Similar to our in vitro analysis of pellet cultures, we detected high levels of COL10 in the implants from the MSCs group, which was not seen in the other experimental groups. Moreover, the newly formed tissue (N) in the Defect group, as well as implanted tissue (I) in the MSCs groups, expressed high levels of COL1, implying the formation of fibrous tissues. In contrast, COL1 was not detected in either native host cartilage tissues (H) or implanted tissue in the iMPCs group. Lastly, most regenerated tissues were from implanted cells (Figure S12). These results collectively suggest the superior potential of iMPCs over MSCs in repairing chondral defects in the knee joint.

### 3.6 Phosphorylation of Smad1/5 and Smad2/3 during the chondrogenesis of MSCs and iMPCs

Having demonstrated that optimal chondrogenesis by iMPCs and MSCs was induced by a different set of stimulatory growth factors, we next explored the mechanism that resulted in such difference. Phosphorylation of Smad2/3 (P‐Smad2/3) has been previously shown to be associated with chondrogenesis. As TGFβ3 alone was not able to induce iMPC chondrogenesis, our first hypothesis was that TGFβ3 treatment could not phosphorylate Smad2/3 in iMPCs.

**FIGURE 7:**
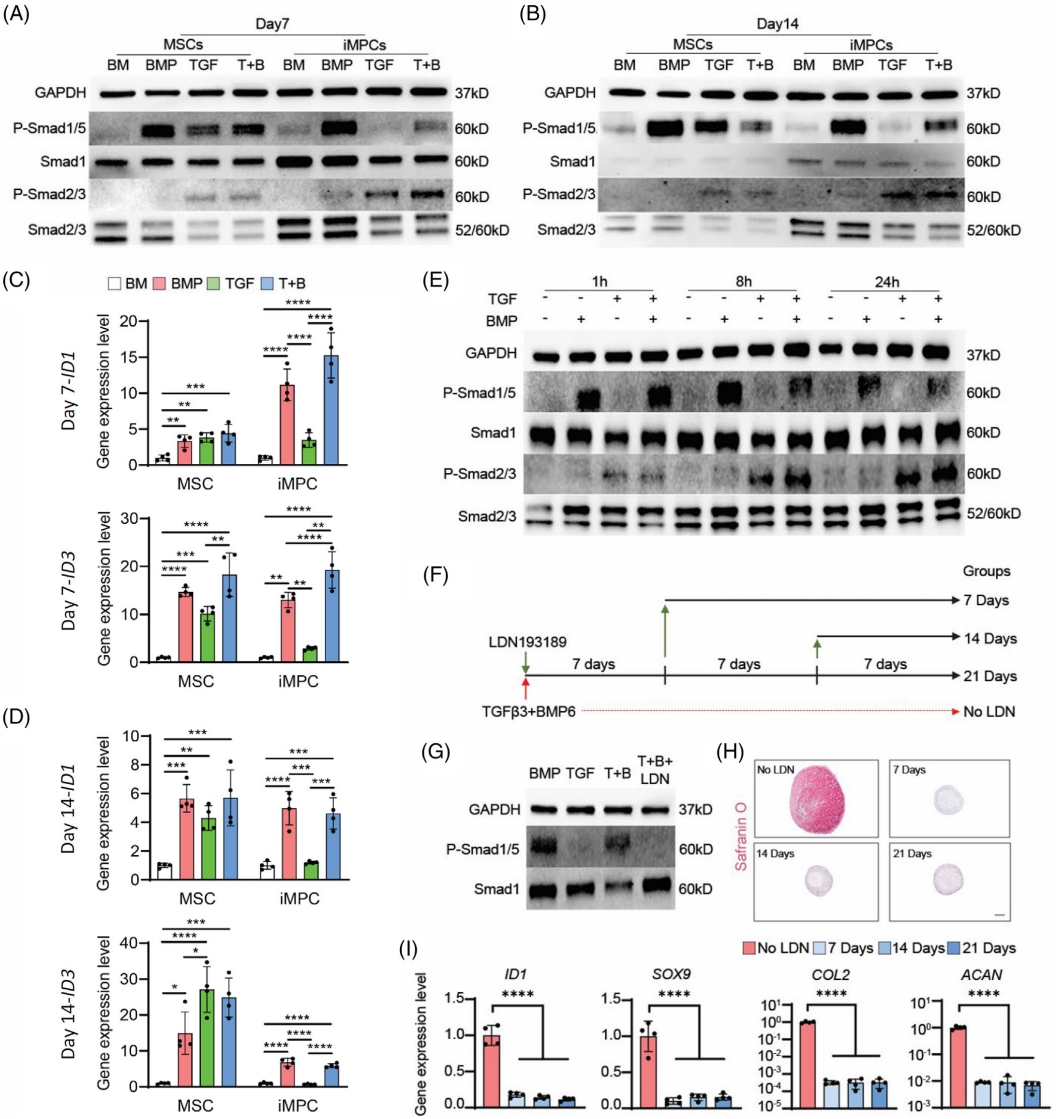
Assessment of the phosphorylation of Smad1/5 and Smad2/3 in MSCs or iMPCs after different treatments. MSCs and iMPCs were formed into pellets first and then subjected to chondrogenic culture in basic chondrogenic medium (BM group), BM with TGFβ3 (TGF group), BM with BMP6 (BMP group) or BM with both TGFβ3 and BMP6 (T+B group) for 7 or 14 days. (A and B) Levels of P‐Smad1/5 and P‐Smad2/3 on day 7 (A) or day 14 (*N* = 3) (B) were examined with Western blot. (C–D) Expression levels of *ID1* and *ID3* in MSCs or iMPCs on days 7 and 14 after chondrogenic culture (*N* = 4). Data were normalized to that in the respective BM group. One‐way ANOVA followed by Tukey's multiple comparisons test was carried out. (E) iMPC pellets were cultured in BM supplemented with different growth factors for 7 days and then starved for 24 h. After fresh medium was added, the phosphorylation of Smad1/5 and Smad2/3 at 1, 8 and 24 h was examined by Western blot (*N* = 3). (F) The timeline of introducing and withdrawing BMP inhibitor (LDN193189, LDN) during the chondrogenesis of iMPCs. (G) The function of LDN was confirmed by examining P‐Smad1/5 level with Western blot on day 7 (*N* = 3). (H) Safranin O staining to assess cartilage formation in iMPC cultures without (No LDN group) or with 7, 14 and 21 days of LDN treatment. Scale bar: 200 μm. (I) Expression levels of *ID1*, *SOX9*, *COL2*, and *ACAN* in cartilage tissues created by iMPCs without (No LDN group) or with at 7, 14 and 21 days of LDN treatment. Data were normalized to that in No LDN group (set as 1) (*N* = 4). One‐way ANOVA followed by Tukey's multiple comparisons test was carried out. *p < .05; **p < .01; ***p < .001; ****p < .0001

To test this hypothesis, we examined levels of P‐Smad2/3 in MSC and iMPC pellet cultures stimulated with TGFβ3 and/or BMP6. Interestingly, TGFβ3 induced a higher level of P‐Smad2/3 in iMPCs than in MSCs (Figure 7A,B and Figure S13), which was out of our expectation.

As previous studies on MSCs suggested that phosphorylation of Smad1/5 is also needed for MSC chondrogenesis,^30^ we thus examined the level of P‐Smad1/5 in all groups. As shown in Figure 7A (day 7 after chondrogenesis), Figure 7B (day 14 after chondrogenesis) and Figure S13, both BMP6 and TGFβ3 induced the phosphorylation of Smad1/5 in MSCs, which was confirmed by the increased expression of inhibitor of DNA binding 1 (*ID1*) and inhibitor of DNA binding 3 (*ID3*) (Figure 7C,D). In contrast, TGFβ alone was not able to induce the phosphorylation of Smad1/5 in iMPCs (Figure 7A–D). As the phosphorylation process could be transient, we further investigated this process in iMPCs at 1, 8 and 24 h after adding fresh medium (Figure 7E). Similar to the results shown in Figure 7A,B, TGFβ alone was able to induce high levels of P‐Smad2/3, but not P‐Smad1/5, in iMPCs.

### 3.7 Need for phosphorylation of Smad1/5 in iMPC chondrogenesis

Given that the phosphorylation of both Smad1/5 and Smad2/3 was observed in the groups that also displayed robust chondrogenic outcomes, we hypothesized that phosphorylation of both Smad1/5 and Smad2/3 is required for successful chondrogenesis of iMPCs. It has been well known that activation of Smad2/3 is necessary for chondrogenesis, so we examined the necessity of Smad1/5 in iMPC chondrogenesis via blocking Smad1/5 pathway with LDN193189 (LDN). Specifically, LDN193189 was introduced into iMPC culture at day 0, with the treatment lasting 7, 14 or 21 days (Figure 7F). LDN treatment successfully blocked the activation of Smad1/5 (Figure 7G) and upregulation of *ID1*(Figure 7I). Meanwhile, *COL2, SOX9* and *ACAN* were significantly suppressed in all LDN193189‐treated groups (Figure 7I), and no GAG deposition was found in iMPC pellet culture after LDN193189 treatment (Figure 7H). In contrast, robust iMPC chondrogenesis was observed in the control (No LDN) group. The results clearly demonstrated that the activation of Smad1/5 is necessary for successful chondrogenesis of iMPCs.

## 4 DISCUSSION

Concomitant chondrocytic hypertrophy, a phenotype that is not seen in healthy hyaline cartilage, is often accompanied with MSC chondrogenesis. In recent years, many researchers have tested iPSCs as a new cell source for regenerating articular cartilage.^7^, ^31^, ^32^, ^33^, ^34^, ^35^, ^36^ In this study, we side‐by‐side compared the quality of cartilage derived from hMSCs and iMPCs and examined their reparative capacity in a rodent animal model. In addition, we conducted a mechanistic study to understand why MSCs and iMPCs respond differently to chondro‐induction factors.

There are different methods to induce iPSC chondrogenesis. In this study, we adopted a technically straightforward protocol that has been previously reported, in which MSC‐like progenitor cells (iMPCs) are directly generated from iPSC culture without undergoing the process of forming an embryoid body.^15^, ^20^, ^37^ In the trilineage differentiation assay that is typically used to characterize MSCs, we observed the minimal adipogenesis and weak chondrogenic potential of iMPCs. A similar observation has been reported independently from several groups,^15^, ^37^, ^38^, ^39^ including iPSC‐derived MSCs that were created through the embryoid body outgrowth method.^15^ A possible explanation is that iMPCs have inherent limitations in generating cartilage or adipose tissues.^37^ However, using another type of adipogenic medium,^40^ we observed robust adipogenesis from iMPCs. In addition, by supplementing BMPs in the conventional TGFβ‐containing chondrogenic medium, iMPCs were also able to undergo robust chondrogenesis. Therefore, the difference of growth factors among differentiation protocols might be decisive for iPSCs‐derived progenitor cells.

To procure sufficient cell numbers for cartilage tissue engineering, MSCs often undergo significant in vitro expansion. Higher cell passage numbers often correspond with a decrease in cell differentiation capacity.^41^, ^42^ In this study, we expanded iMPCs up to passage 7 and examined their capacity in generating colonies and differentiating into osteoblast, chondrocyte and adipocyte‐like cells. As shown in Figure 1, it is clear that increased expansion rapidly decreased the stemness of iMPCs. For example, at passage 7, only several colonies were seen from iMPCs culture. A previous study examined the CFU‐forming capacity of P3, P5 and P7 primary MSCs and found that around 41% of P3 cells formed colonies, which decreased to 23% and 19.7% at P5 and P7, respectively.^43^ In our study, only 1%–2% of P7 iMPCs formed colonies. Regarding the chondrogenic differentiation capacity, Sun et al. recently reported that human bone marrow‐derived MSCs at P3 had better chondrogenic ability compared to cells at P5 and P7,^44^ which was similar to what we showed in Figure 1F.

When examining the expression profile of surface makers, we noticed that primary MSCs contain more CD105‐positive cells than iMPCs. Interestingly, a higher CD105 expression has been shown to associate with higher chondrogenesis.^45^, ^46^, ^47^ We also examined the expression level of CD105 among different passages of iMPCs, and the results showed that the positive ratios of CD105 in P5 and P7 cells were lower than that in P3 cells. However, it was previously reported that expression of CD105 in MSCs increased during passaging.^48^ Therefore, the exact role of CD105 in MSCs and iMPCs requires careful study.

For the chondrogenesis of MSCs, TGFβ has been a critical component in conventional chondrogenic differentiation medium. Most previous publications and our current study clearly indicated that TGFβ alone is not able to induce iMPC chondrogenesis.^15^, ^16^, ^17^, ^37^ There was only one research reporting that TGFβ alone could also induce iMPC chondrogenesis,^12^ which may be associated with the use of matrigel during the mesenchymal differentiation of iPSCs. In general, BMP2, BMP4 or TGFβ combined with BMPs and other growth factors can result in robust cartilage formation from the whole population of iMPCs.^12^, ^34^, ^35^, ^49^, ^50^ For example, Guzzo et al. reported that micromass culture of iPSC‐derived progenitor cells treated with BMP2, not TGFβ1, shows positive Alcian Blue staining and the upregulation of cartilage‐related genes.^17^ In another study, BMP4 was used to enhance the chondrogenic behaviour of iMPCs, although undesired hypertrophic phenotype occurred after treatment.^19^ To the best of our knowledge, the comparison of these chondro‐inducing methods from different studies was not performed, raising the question of which method is the best. Therefore, we tested different BMPs, also several growth factors from TGFβ super family, including TGFβ, growth/differentiation factors (GDF)5, GDF9, Nodal, Activin A and their combinations, for iMPC chondrogenesis (Figure 2 and Figures S5–S7). A surprising finding in this study is that BMP4 induces robust iMPC chondrogenesis without the need of TGFβ. BMP4‐induced chondrogenesis had also been previously reported. For example, BMP4 alone was sufficient to induce chondrogenesis of mesangial cells.^51^ In the limb bud culture, the addition of BMP4 to the media caused significant increases in the expression levels of chondrogenic genes.^52^ In another relevant study, BMP2 or 7 displayed significantly higher chondro‐induction potential than TGF‐β1 in synovial cell culture.^53^ As discussed above, hypertrophy is one of the major concerns in applying MSCs for chondral repair. We carefully evaluated the hypertrophy level in iMPC‐derived cartilage when comparing different chondro‐induction methods. BMP4 alone resulted in a high level of chondrogenesis, which however induced the deposition of IHH, a representative hypertrophy marker. In comparison, the combination of TGFβ3 and BMP6 resulted in robust hyaline‐like cartilage formation in both in vitro and in vivo studies.

Currently, the regulatory network that dictates the initiation and maintenance of hypertrophy is not clear, but RUNX2 might be an important regulator.^54^, ^55^ For example, a recent study showed that suppressing RUNX2 with shRNA in MSCs significantly reduced the expression of *COL10* after chondrogenesis.^56^ In our study, we observed significantly lower expression of RUNX2 during iMPC chondrogenesis since day 7 when compared to MSCs. After the addition of BMP6, we found that both iMPC and MSC did not show the increased expression of RUNX2 (Figure 4E), which indicates that BMP6 may not be the activator for the hypertrophic phenotype in iMPCs. Ko et al. also reported that both naïve iPSCs and chondrogenic differentiated iPSCs displayed significantly lower expression of RUNX2 than undifferentiated MSCs and differentiated MSCs.^57^ These results collectively indicate that iMPCs have inherently low RUNX2 expression, which might limit the hypertrophic and osteogenic transition during chondrogenesis.

We then investigated the role of BMP6 in iMPC chondrogenesis. Previously, BMP6 has been used as an enhancing factor for the chondrogenesis of adipose‐derived MSCs or other subpopulations of MSC.^58^, ^59^ A study from Hennig et al. indicated that BMP6 functioned through promoting the expression of ALK5 in adipose‐derived stem cells. However, this should not be the case for iMPCs, as iMPCs expressed a high level of ALK5 (Figure S14). We then directed our attention to the activation of Smad2/3 and Smad1/5. Activation of Smad2/3 is required to induce chondrogenesis in MSCs through activating SOX9, a key factor in early chondrogenesis.^60^ In contrast, the knockdown of Smad3 significantly inhibited TGFβ‐induced chondrogenic differentiation.^61^ However, our results indicated that TGFβ alone sufficiently resulted in high level of P‐Smad2/3, which did not lead to an early expression of SOX9 and further chondrogenesis (Figure 4E). Interestingly, the addition of BMP6, which only activated Smad1/5, not Smad2/3, resulted in a higher expression of SOX9 and better chondrogenesis of iMPCs (Figures 4E and 7A,B). Of note, TGFβ induced the phosphorylation of both Smad2/3 and Smad1/5 and robust chondrogenesis in MSC culture. These results raised our interest in understanding the role of Smad1/5 in chondrogenesis. First, we noticed that the quality of total Smad1 is higher in iMPCs than in MSCs. And, Smad1/5 is also critical for cartilage development.^62^ Smad1/5 knock‐out in mice has demonstrated severe chondrodysplasia, and the loss of BMP‐Smad1/5 signaling causes the reduction of chondrocyte proliferation and elevated apoptosis.^54^ We also confirmed the importance of P‐Smad1/5 in iMPC chondrogenesis by using LDN193189 to block BMP/Smad1/5 signaling (Figure 7G–I). Actually, similar results were previously reported in a study using MSCs.^30^ Therefore, we confirmed that activation of P‐Smad1/5, at least during the initiation stage, is necessary for successful chondrogenesis. Of note, P‐Smad1/5 has also been considered as a key signaling pathway leading to chondrocyte hypertrophy.^54^, ^63^, ^64^ In our study, the addition of BMP6 successfully activated the Smad1/5 in iMPCs; however, it did not induce high expression of RUNX2 and other hypertrophic markers, especially compared to BMSCs. To the best of our knowledge, there are no studies reporting how TGFβ‐Smad and BMP‐Smad signaling pathways interact during iMPC chondrogenesis and hypertrophy. We believe the role of P‐Smad1/5 in generating hyaline cartilage from stem cells needs to be precisely tuned. For example, activation of Smad1/5 is necessary at the early stage of chondrogenesis, which however needs to be deactivated to suppress hypertrophic transition after chondrogenesis is established.

There are some limitations in this study that need to be addressed in the future. First, MSCs used in this study were from old donors, because they were isolated from the surgical waste of total joint replacement. In the future, it will be interesting to compare iMPCs to MSCs that are derived from fetus tissues. Second, the addition of BMP6 induced the significant phosphorylation of Smad1/5 in iMPCs, which however did not result in high expression of RUNX2 and hypertrophy. The mechanism underlying the maintenance of low RUNX2 level in iMPCs has not been fully understood. Third, a rat model was used in this study, which demonstrated the capacity of iMPC‐derived cartilage in maintaining the hyaline cartilage‐like phenotype in osteochondral defects in immunodeficient rats. In the future, a preclinical model with normal immune system can be used to examine the reparative capacity of iMPC‐derived cartilage in repairing chondral defects, which will be more clinically relevant. Lastly, the underlying mechanism that TGFβ alone could not activate Smad1/5 still needs future study to explore. Yet, the current results laid the groundwork to further understand the chondrogenic behaviour of iMPCs.

## 5 CONCLUSION

Human iMPCs created from iPSCs are promising cell sources for cartilage regeneration. Here, we concluded an optimal induction method to generate hyaline cartilage‐like tissue from iPSC‐derived iMPCs. Through comprehensive in vitro and in vivo studies, we demonstrated that iMPCs represent a better cell source than primary MSCs in regenerating hyaline cartilage. In the mechanistic study, we revealed the critical role of Smad1/5 in initiating chondrogenesis of both MSCs and iMPCs, and elucidated the mechanism underlying BMP6‐augmented iMPC chondrogenesis.

## CONFLICT OF INTEREST

The authors declare that there is no conflict of interest.

## Supporting information

Supporting material

Supporting material

Supporting material

Supporting material

Supporting material
