# Supplementary material for: Differences in the intrinsic chondrogenic potential of human mesenchymal stromal cells and iPSC‐derived multipotent cells
Source: Clin Transl Med. 2022 Dec 19;12(12):e1112. doi: 10.1002/ctm2.1112 (PMC9763539; doi:10.1002/ctm2.1112)
Supplement: Supplementary file 1 — Supporting material [file CTM2-12-e1112-s001.docx]

**Supplementary Information**

**Differences in the intrinsic chondrogenic potential of human mesenchymal stromal cells and iPSC-derived multipotent cells**

Shiqi Xiang^1,2^, Zixuan Lin^1^, Meagan J. Makarcyzk^1,3^, Kanyakorn Riewruja^1,4^, Yiqian Zhang^1^, Xiurui Zhang^1^, Zhong Li^1^, Karen L. Clark^1^, Eileen Li^1^, Silvia Liu^5^, Tingjun Hao^1^, Madalyn R. Fritch^1^, Peter G. Alexander^1,6^, Hang Lin^1,3,6^*

^1^Department of Orthopaedic Surgery, University of Pittsburgh School of Medicine; 450 Technology Drive, Rm 217, Pittsburgh, Pennsylvania, 15219, USA.

^2^ Department of Orthopaedics, The Second Xiangya Hospital, Central South University, Changsha, Hunan, PR China

^3^Department of Bioengineering, University of Pittsburgh Swanson School of Engineering; 450 Technology Drive, Rm 217, Pittsburgh, Pennsylvania, 15219, USA.

^4^Osteoarthritis and Musculoskeleton Research Unit, Faculty of Medicine, Chulalongkorn University, King Chulalongkorn Memorial Hospital, Thai Red Cross Society, Bangkok 10330, Thailand

^5^Department of Pathology, University of Pittsburgh School of Medicine; S-406 Biomedical Science Tower, 203 Lothrop Street, Pittsburgh, PA 15261, USA

^6^McGowan Institute for Regenerative Medicine, University of Pittsburgh School of Medicine; 450 Technology Drive, Rm 217, Pittsburgh, Pennsylvania, 15219, USA.

Running Title: Hyaline Cartilage from iPSCs

*Correspondence:

Hang Lin, PhD

450 Technology Drive, Room 217,

Pittsburgh, PA, 15219, USA

Phone: 412-624-5503

Fax: 412-624-5544

Email: [hal46@pitt.edu](mailto:hal46@pitt.edu)

**Supplementary Figure S1**


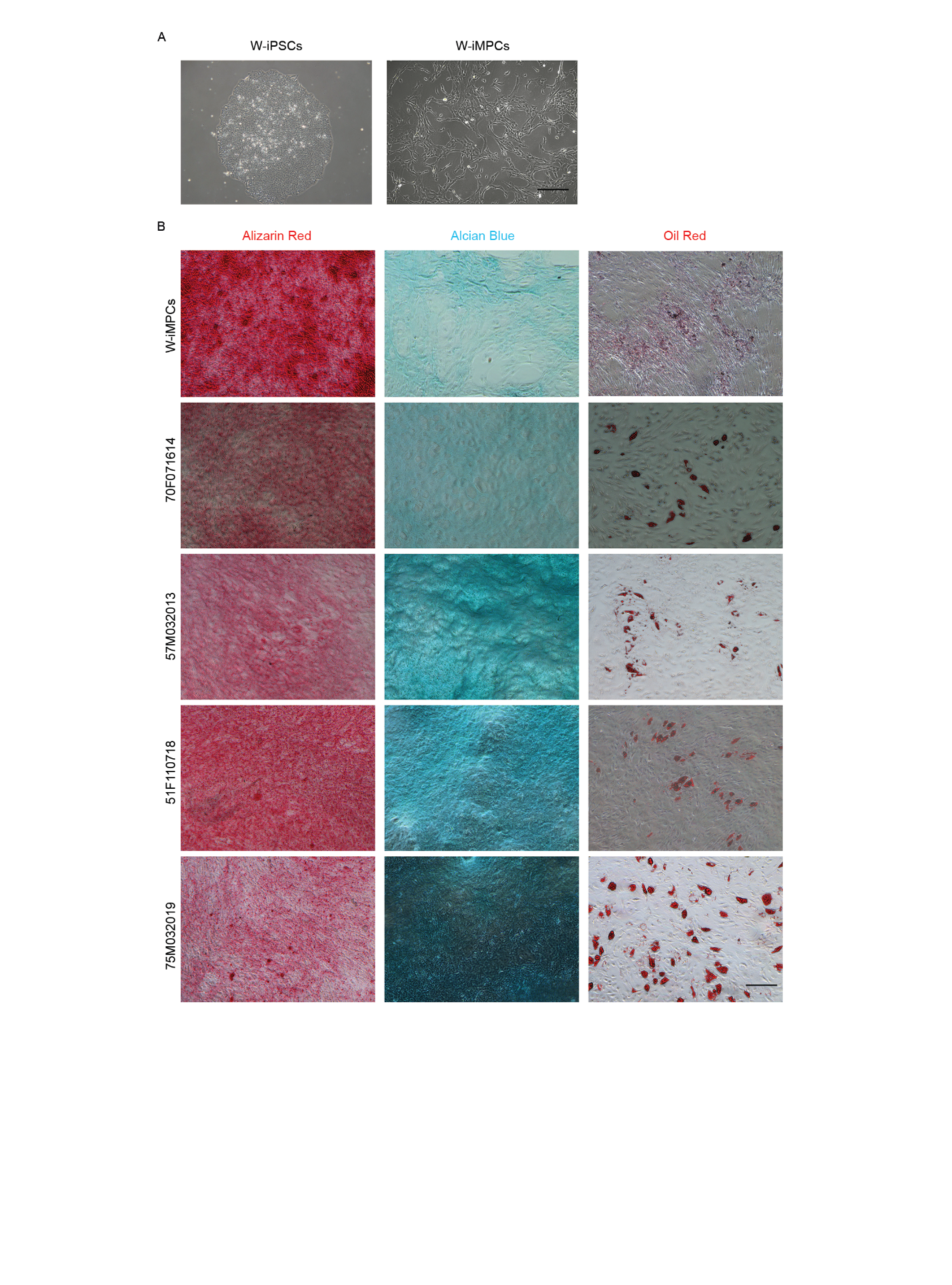


**Supplementary Figure S1. Characterization of iMPCs from W-iPSCs and MSCs from four donors. (A)** Representative cell morphology of W-iPSCs and iMPCs derived from W-iPSCs (W-iMPCs). Scale bar: 200 μm. **(B)** Alizarin Red staining, Alcian blue staining and Oil red staining for W-iMPCs and MSCs after 21 days differentiation. Scale bar: 200 μm.

**Supplementary Figure S2**


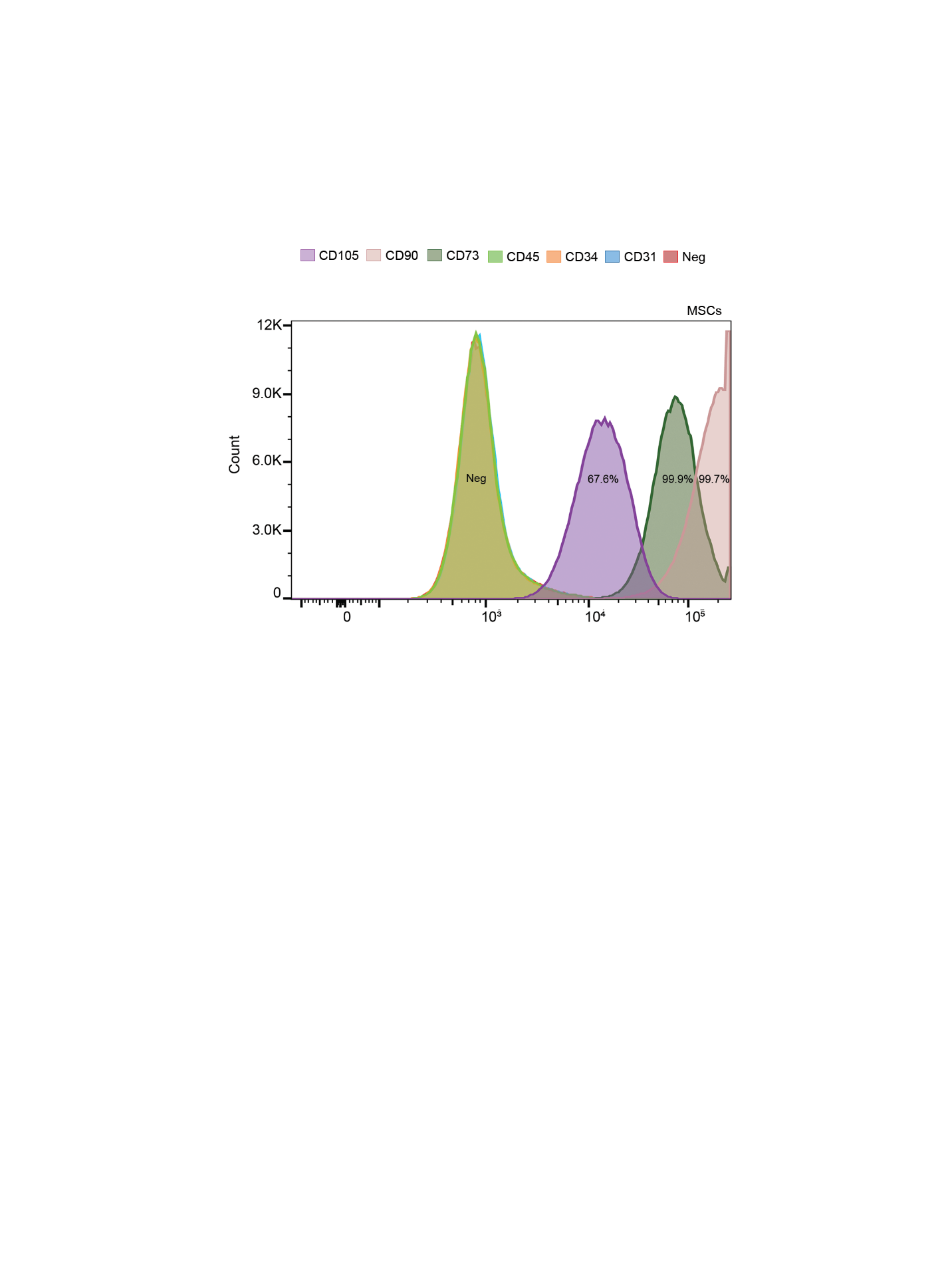


**Supplementary Figure S2. Flow cytometry analysis of surface marker expression in MSCs.**

**Supplementary Figure S3**

**
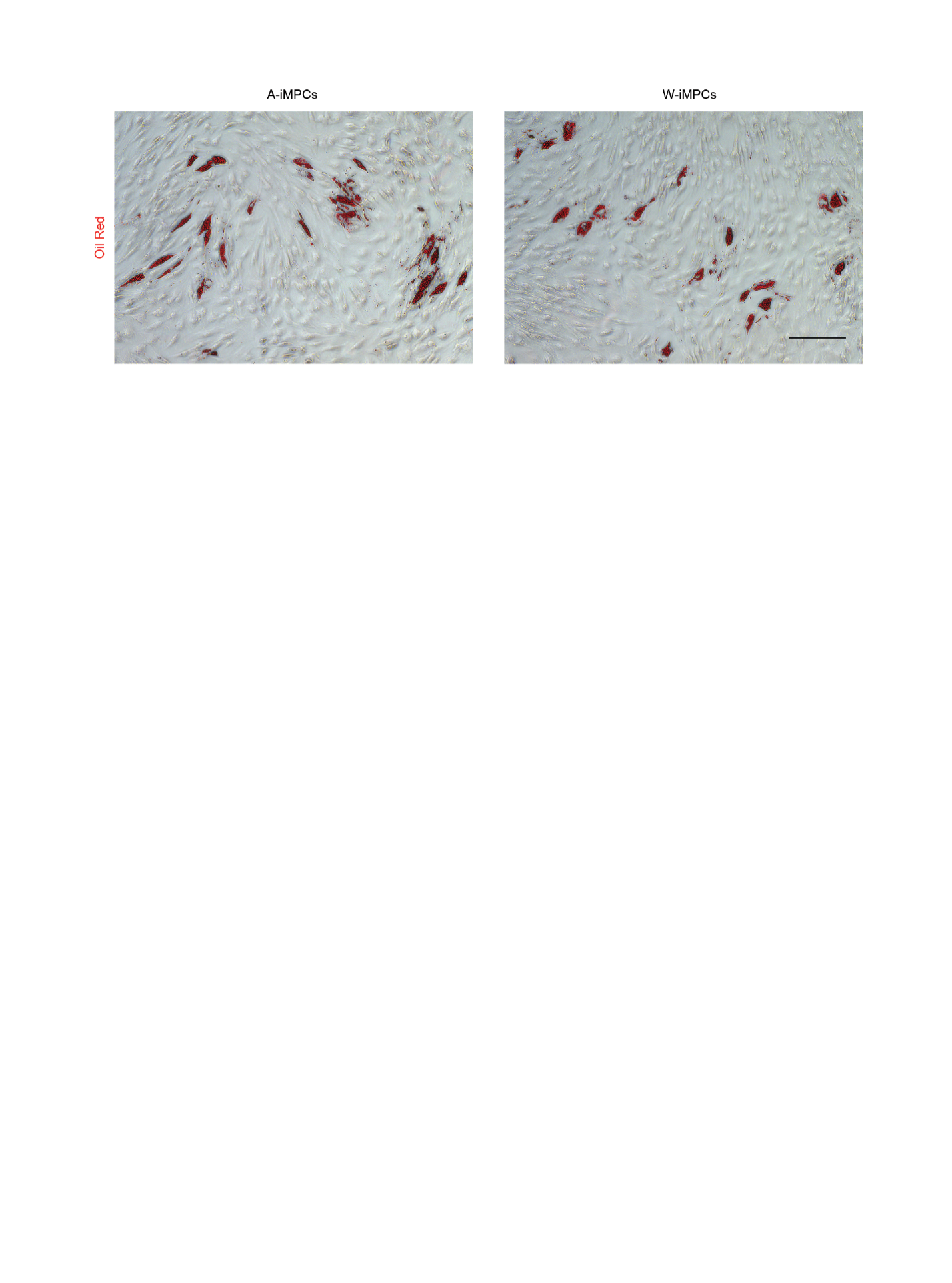
**

**Supplementary Figure S3. New protocol to induce iMPC adipogenesis.** After 21 days culture in another type of adipogenic medium [29], iMPCs derived from A-iPSCs (A-iMPCs) and W-iPSCs (W-iMPCs) were stained with Oil red. Scale bar: 100 μm.

**Supplementary Figure S4**


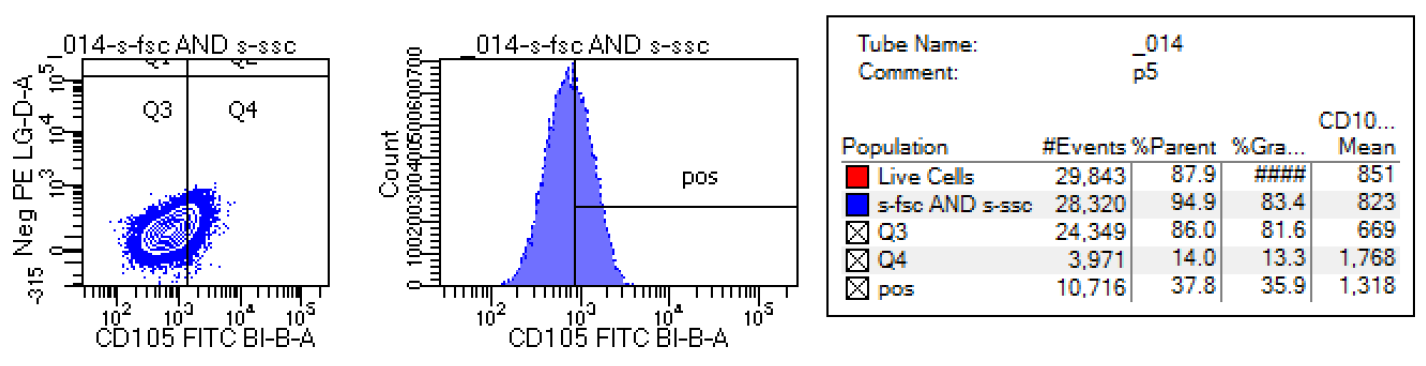


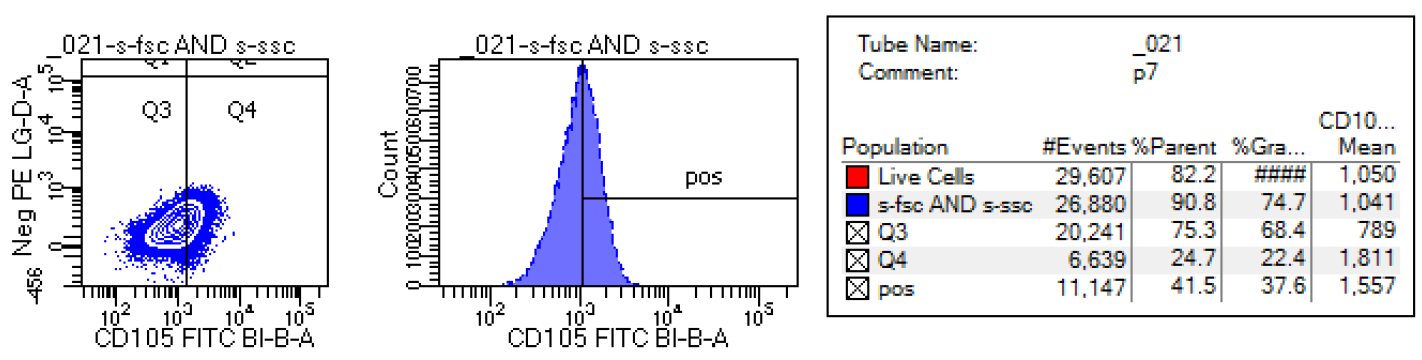


**Supplementary Figure S4. Flow cytometry analysis of CD105 expression in P5 and P7 iMPCs.**

**Supplementary Figure S5**


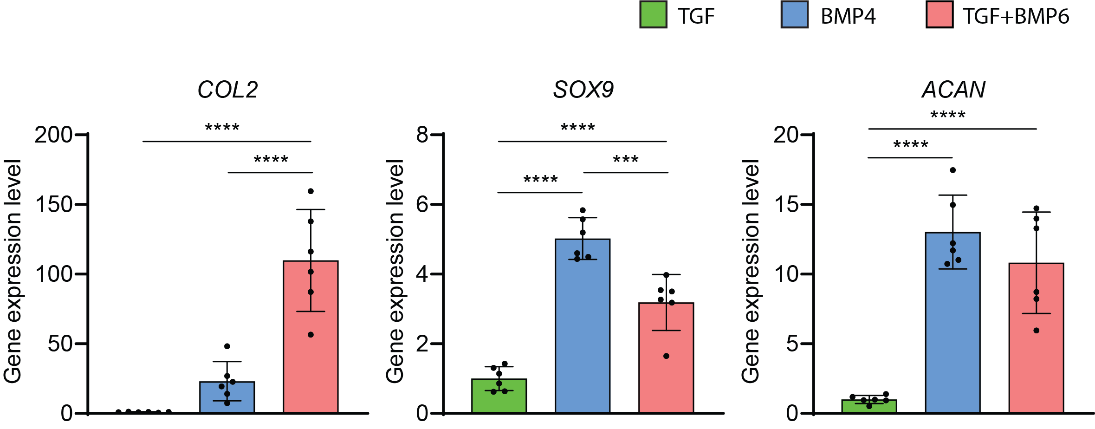


**Supplementary Figure S5. Chondrogenesis of iMPCs (derived from C-iPSC) after 21-day culture in basic chondrogenic medium supplemented with different growth factors.** Relative expression levels of chondrogenesis-related markers (SOX9, COL2 and ACAN) after 21 days chondro-induction were shown. Data were normalized to that in TGF group (set as 1) (N=6). One-way ANOVA followed by Tukey’s multiple comparisons test was carried out. ***, p<0.001; ****, p<0.0001.

**Supplementary Figure S6**

**
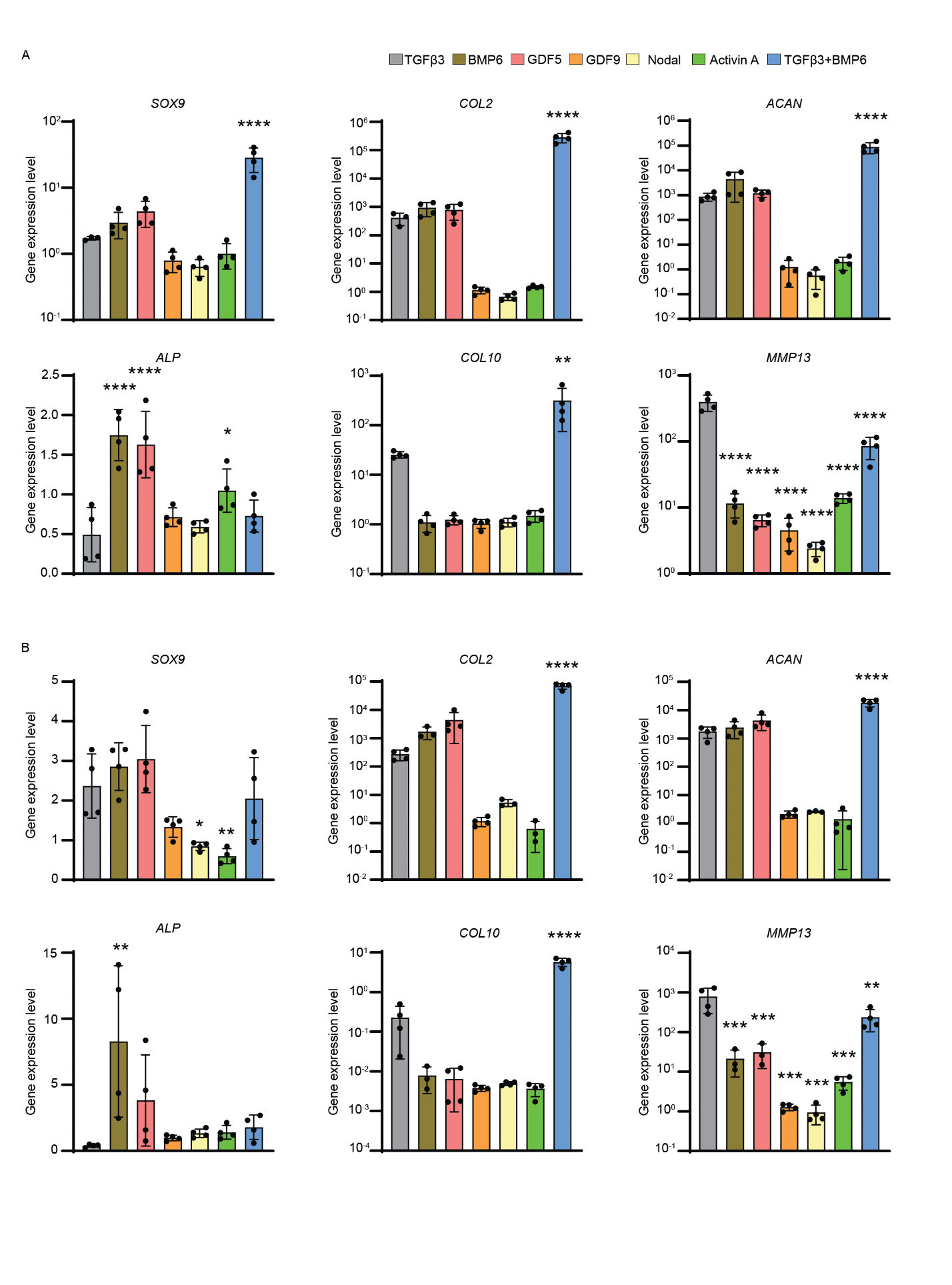
Supplementary Figure S6. Assessment of A-iMPC chondrogenesis after 21-day culture in basic chondrogenic medium (BM) supplemented with different growth factors.** Relative expression levels of chondrogenesis-related markers (*SOX9, COL2* and *ACAN*). Data were normalized to that in BM group (set as 1). BM is not shown in the figure. (N= 4) One-way ANOVA followed by Dunnett's multiple comparisons test. ****, p<0.0001.

**Supplementary Figure S7**

**
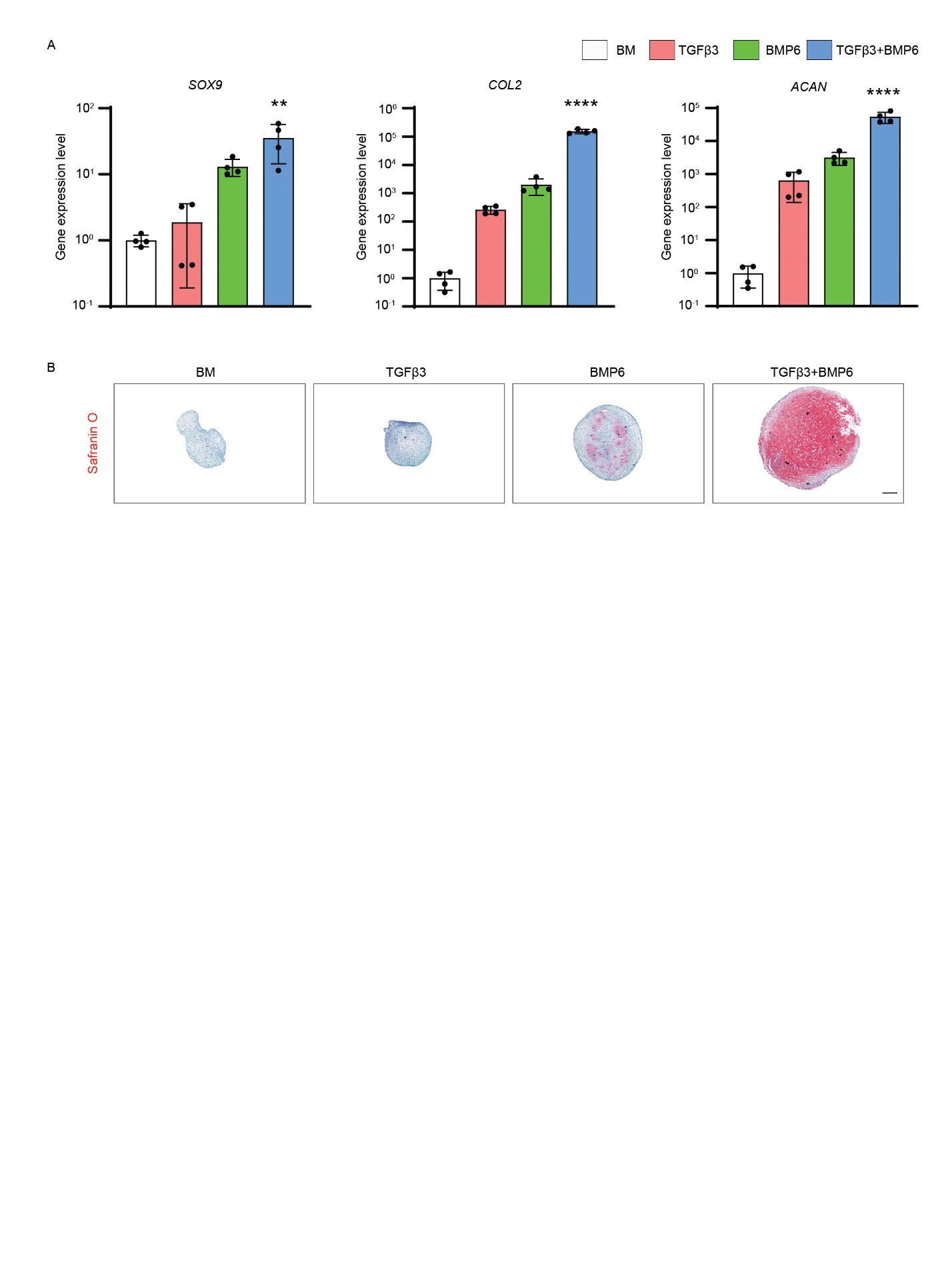
**

**Supplementary Figure S7. Chondrogenesis of W-iMPCs after 21-day culture in basic chondrogenic medium (BM) supplemented with different growth factors. (A)** Relative expression levels of chondrogenesis-related markers (*SOX9, COL2* and *ACAN*) after 21 days chondro-induction. Data were normalized to that in BM group (set as 1) (N=4). One-way ANOVA followed by Dunnett's multiple comparisons test. (BM group as the control) **, p<0.01; ****, p<0.0001. **(B)** Safranin O staining. Scale bar: 200 μm.

**Supplementary Figure S8**

**
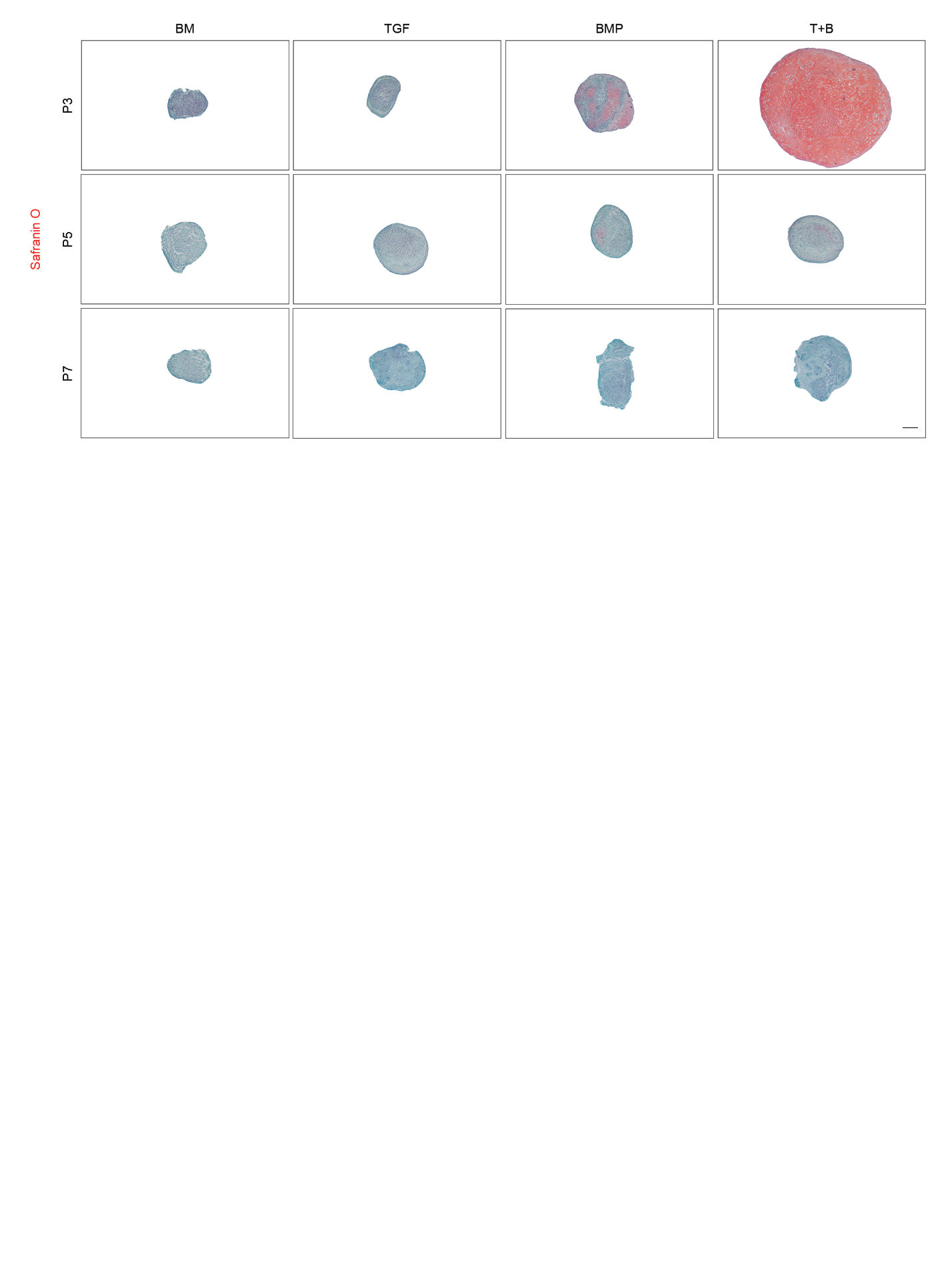
**

**Supplementary Figure S8. Assessment of chondrogenic potential of iMPCs after being passaged 3 (P3), 5 (P5) and 7 (P7) times.** Safranin O staining was used to examine GAG production after 21-day culture in basic chondrogenic medium (BM) supplemented with different growth factors. Scale bar: 200 μm.

**Supplementary Figure S9**


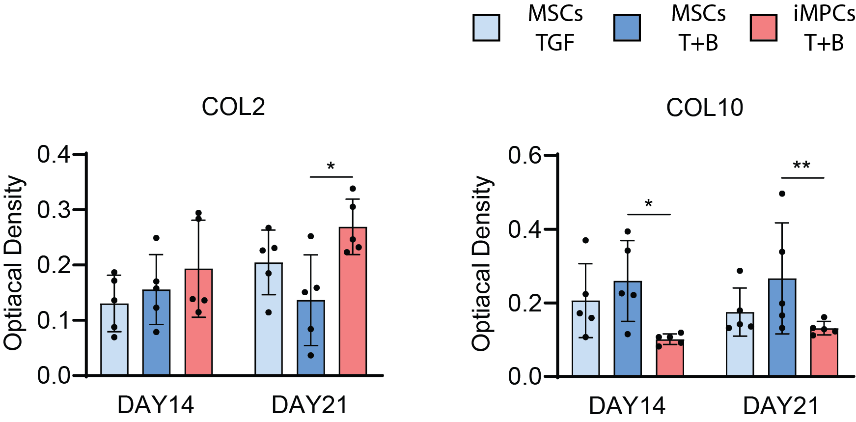


**Supplementary Figure S9. COL2 and COL10 IHC quantification for Figure 5 G&H.** Quantitative optical density of IHC staining for COL2, COL10. (N=5), One-way ANOVA followed by Tukey’s multiple comparisons test was carried out. *, p<0.05; **, p<0.01.

**Supplementary Figure S10**


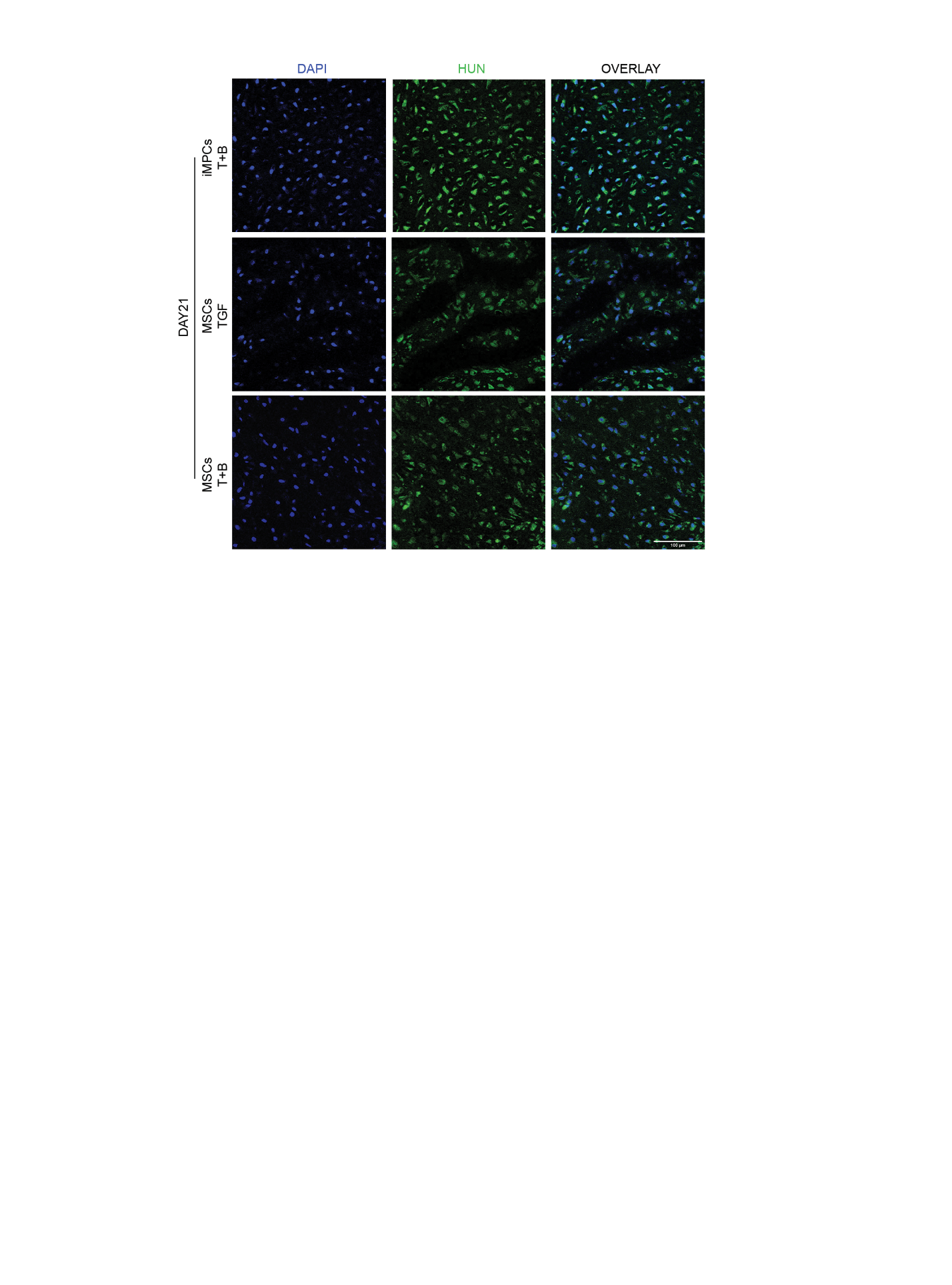


**Supplementary Figure S10. Immunofluorescence staining for MSCs or iMPCs- derived cartilage tissue after subcutaneous implantation.** DAPI (blue), nucleus; HUN (green), human nuclear antigen. Scale bar: 100 μm.

**Supplementary Figure S11**


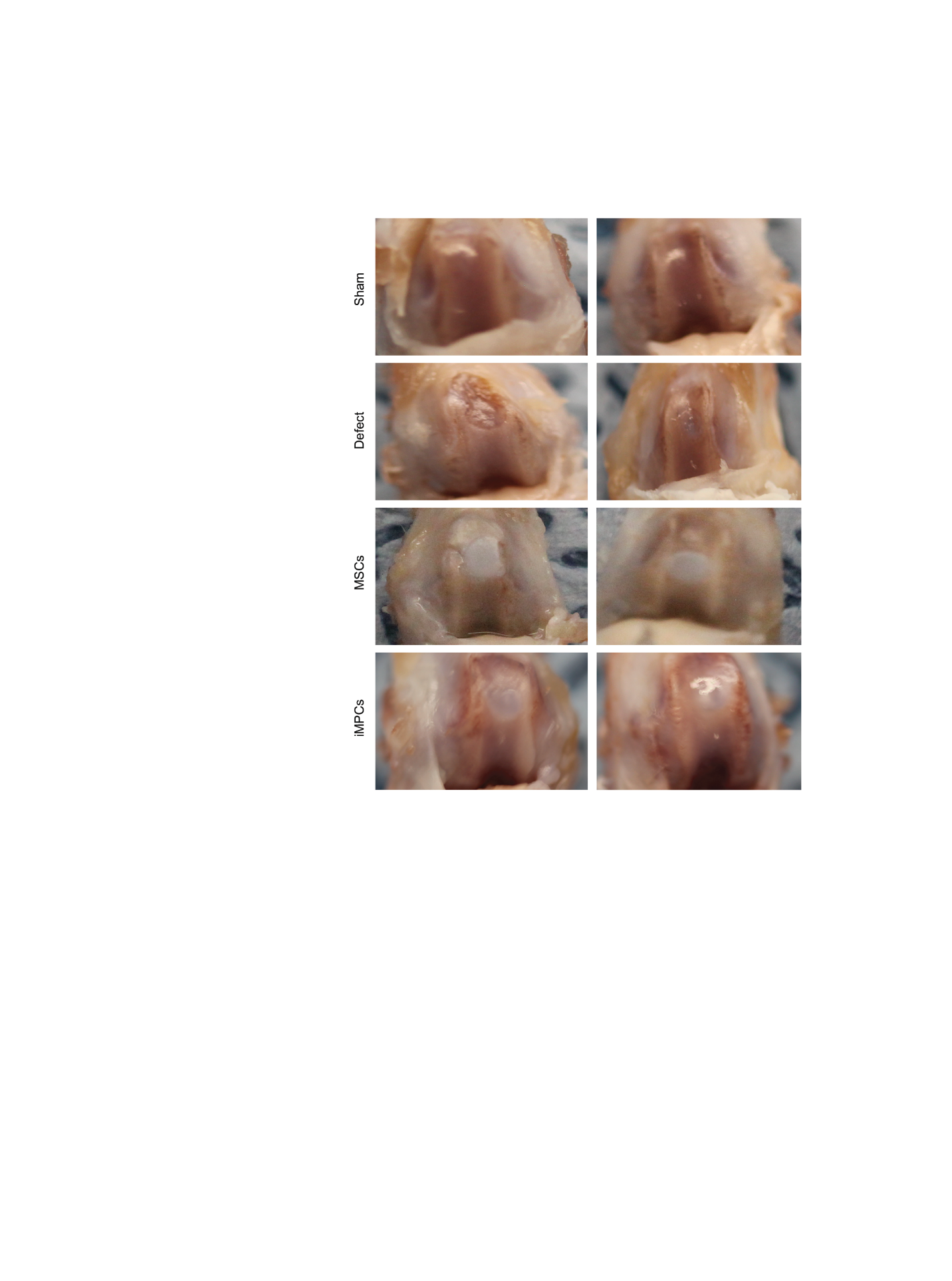


**Supplementary Figure S11. Macro-appearance of the defects in four groups.**

**Supplementary Figure S12**


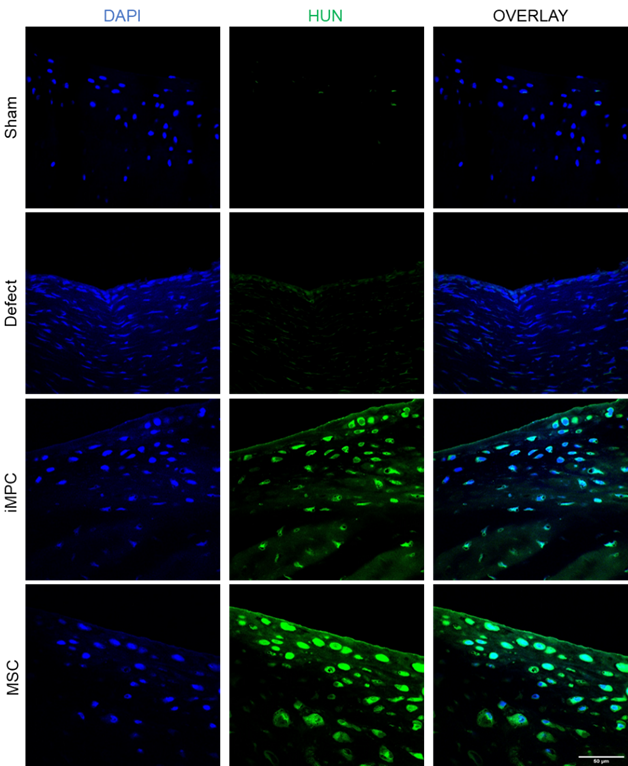


**Supplementary Figure S12. Immunofluorescence staining for tissues in osteochondral defect in rat.** DAPI (blue), nucleus; HUN (green), human nuclear antigen. Scale bar: 50 μm.

**Supplementary Figure S13**


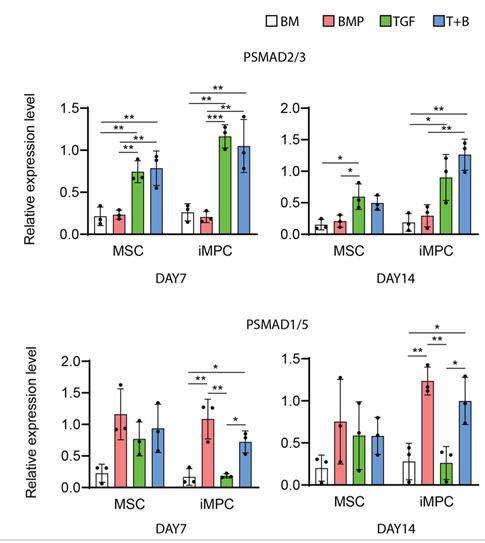


**Supplementary Figure S13. Quantification of the protein levels of pSmad2/3 and pSmad1/5 from Figure 7A, B.** Band intensities were quantified by image J and then normalized to GAPDH. (N=3). One-way ANOVA followed by Tukey’s multiple comparisons test was carried out. *, p<0.05; **, p<0.01; ***, p<0.001.

**Supplementary Figure S14**


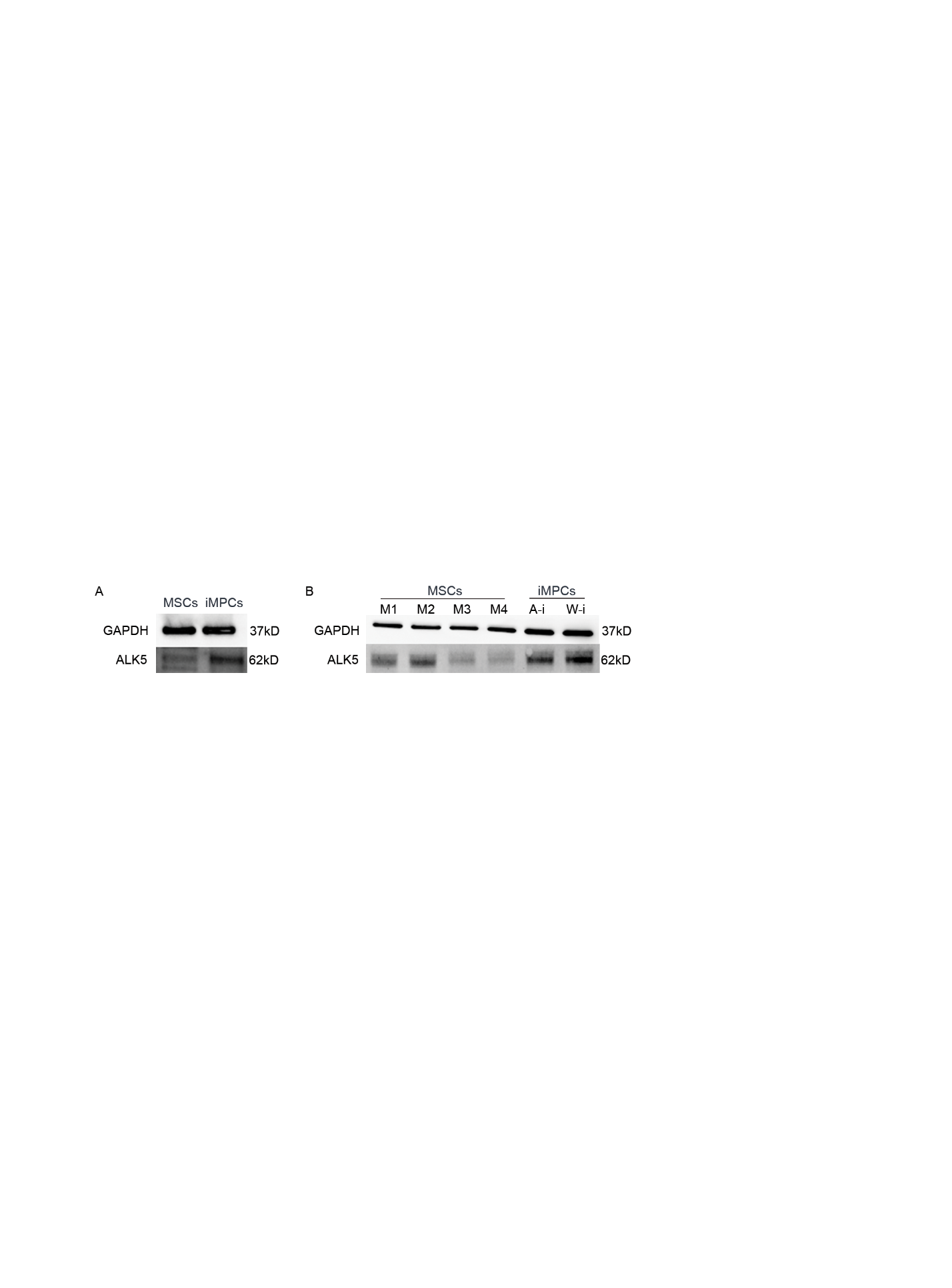


**Supplementary Figure S14. Assessment of ALK5 level in MSCs and iMPCs.**  (A) Levels of ALK5 in pooled MSCs and iMPCs at passage 3 were examined by western blot. (B) The protein levels of ALK5 in MSCs isolated from four patients (M1-4), and in iMPCs generated from A-iPSCs (A-i) or W-iPSCs (W-i).

**Supplementary Figure S15**


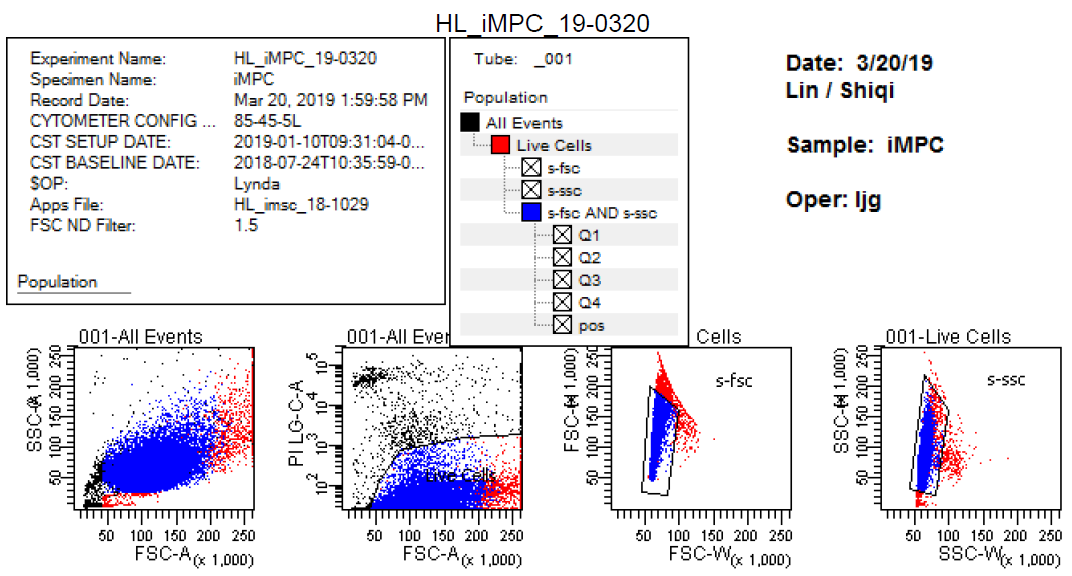


**Supplementary Figure S15. Gating strategy in flow cytometry.** Briefly, the live cells are gated (PI-). Then, FSCH/W and SSCH/W are used for doublet discrimination. From this population, there are dot plots of the PE marker vs FITC markers and histograms of the FITC markers on the Figure.
