# Supplementary material for: Differences in the intrinsic chondrogenic potential of human mesenchymal stromal cells and iPSC‐derived multipotent cells
Source: Clin Transl Med. 2022 Dec 19;12(12):e1112. doi: 10.1002/ctm2.1112 (PMC9763539; doi:10.1002/ctm2.1112)
Supplement: Supplementary file 2 — Supporting material [file CTM2-12-e1112-s002.docx]

**Supplementary Table S****1. Primers for qRT-PCR**

| **Gene** | **Forward primer (5’-3’)** | **Reverse primer (5’-3’)** |
| --- | --- | --- |
| *SOX9* | GGCGGAGGAAGTCGGTGAAGAA | GCTCATGCCGGAGGAGGAGTGT |
| *ACAN* | AGTCACACCTGAGCAGCATC | AGTTCTCAAATTGCATGGGGTGTC |
| *COL2* | GGATGGCTGCACGAAACATACCGG | CAAGAAGCAGACCGGCCCTATG |
| *ALP* | ATCTTTGGTCTGGCCCCCATG | AGTCCACCATGGAGACATTCTCTC |
| *COL10* | CCCTCTTGTTAGTGCCAACC | AGATTCCAGTCCTTGGGTCA |
| *MMP13* | ATGCAGTCTTTCTTCGGCTTAG | ATGCCATCGTGAAGTCTGGT |
| *RUNX2* | GTGATAAATTCAGAAGGGAGG | CTTTTGCTAATGCTTCGTGT |
| *ID1* | CTGCTCTACGACATGAACGG | GAAGGTCCCTGATGTAGTCGAT |
| *ID3* | GAGAGGCACTCAGCTTAGCC | TCCTTTTGTCGTTGGAGATGAC |
| *GAPDH* | CAAGGCTGAGAACGGGAAGC | AGGGGGCAGAGATGATGACC |
| *RPL13A* | CATAGGAAGCTGGGAGCAAG | GCCCTCCAATCAGTCTTCTG |
