# Supplementary material for: Differences in the intrinsic chondrogenic potential of human mesenchymal stromal cells and iPSC‐derived multipotent cells
Source: Clin Transl Med. 2022 Dec 19;12(12):e1112. doi: 10.1002/ctm2.1112 (PMC9763539; doi:10.1002/ctm2.1112)
Supplement: Supplementary file 3 — Supporting material [file CTM2-12-e1112-s004.docx]

**Supplementary Table S2. Antibodies used for flow cytometry (FC), immunofluorescence (IF), Immunohistochemistry (IHC), or Western blot (WB).**

| **Antibody** | **Origin** | **Cat. No** | **Species** | **Assay** | **Dilution** |
| --- | --- | --- | --- | --- | --- |
| FITC Mouse Anti-Human CD31 | BD Biosciences | 557508 | Mouse | FC | 1:4 |
| FITC Mouse Anti-Human CD34 | BD Biosciences | 560942 | Mouse | FC | 1:4 |
| FITC Mouse Anti-Human CD45 | BD Biosciences | 560976 | Mouse | FC | 1:4 |
| FITC Mouse Anti-Human CD73 | BD Biosciences | 561254 | Mouse | FC | 1:19 |
| FITC Mouse Anti-Human CD90 | BD Biosciences | 561969 | Mouse | FC | 1:19 |
| FITC Mouse anti-Human CD105 | BD Biosciences | 561443 | Mouse | FC | 1:19 |
| Anti-Human Nuclear Antigen antibody | Abcam | ab191181 | Mouse | IF | 1:200 |
| Goat Anti-Mouse IgG H&L (Alexa Fluor® 488) | Abcam | ab150113 | Goat | IF | 1:1000 |
| Anti-Human Collagen Type II | MP Biomedicals | SKU 0863171 | Mouse | IHC | 1:100 |
| Anti-Collagen I antibody | Abcam | ab6308 | Mouse | IHC | 1:500 |
| Collagen X Monoclonal Antibody (X53) | Invitrogen | 14-9771-95 | Mouse | IHC | 1:100 |
| Recombinant Anti-Ihh antibody | Abcam | ab52919 | Rabbit | IHC | 1:50 |
| Goat Anti-Mouse IgG H&L (HRP) | Abcam | ab205719 | Goat | IHC/WB | 1:1000 |
| Goat Anti-Rabbit IgG H&L (HRP) | Abcam | ab7090 | Goat | IHC/WB | 1:1000 |
| GAPDH (D16H11) XP® Rabbit mAb | Cell Signaling Technology | 5174S | Rabbit | WB | 1:2000 |
| Smad1 (D59D7) XP® Rabbit mAb | Cell Signaling Technology | 6944s | Rabbit | WB | 1:1000 |
| RUNX2 (D1H7) Rabbit mAb | Cell Signaling Technology | 8486s | Rabbit | WB | 1:1000 |
| Phospho-Smad1/5 (Ser463/465) (41D10) Rabbit mAb | Cell Signaling Technology | 9516s | Rabbit | WB | 1:1000 |
| Recombinant Anti-Smad1 (phospho S463 + S465) antibody | Abcam | ab226821 | Rabbit | WB | 1:1000 |
| Collagen X Recombinant Rabbit Monoclonal Antibody | Invitrogen | MA5-32504 | Rabbit | WB | 1:1000 |
| Recombinant Anti-SOX9 antibody | Abcam | ab185230 | Rabbit | WB | 1:1000 |
| Collagen II Monoclonal Antibody | Invitrogen | MA5-12789 | Rabbit | WB | 1:200 |
| Phospho-Smad2 (Ser465/467) (138D4) Rabbit mAb | Cell Signaling Technology | 3108S | Rabbit | WB | 1:1000 |
| Smad2/3 (D7G7) XP® Rabbit mAb | Cell Signaling Technology | 8685S | Rabbit | WB | 1:1000 |
| Anti-TGF beta Receptor I antibody | Abcam | ab31013 | Rabbit | WB | 1:1000 |
